# A new method for obtaining model-free viscoelastic material properties from atomic force microscopy experiments using discrete integral transform techniques

**DOI:** 10.3762/bjnano.12.79

**Published:** 2021-09-23

**Authors:** Berkin Uluutku, Enrique A López-Guerra, Santiago D Solares

**Affiliations:** 1 Department of Mechanical and Aerospace Engineering, The George Washington University School of Engineering and Applied Science, Washington, District of Columbia, USA

**Keywords:** atomic force microscopy, force spectroscopy, material properties, viscoelasticity

## Abstract

Viscoelastic characterization of materials at the micro- and the nanoscale is commonly performed with the aid of force–distance relationships acquired using atomic force microscopy (AFM). The general strategy for existing methods is to fit the observed material behavior to specific viscoelastic models, such as generalized viscoelastic models or power-law rheology models, among others. Here we propose a new method to invert and obtain the viscoelastic properties of a material through the use of the Z-transform, without using a model. We present the rheological viscoelastic relations in their classical derivation and their *z*-domain correspondence. We illustrate the proposed technique on a model experiment involving a traditional ramp-shaped force–distance AFM curve, demonstrating good agreement between the viscoelastic characteristics extracted from the simulated experiment and the theoretical expectations. We also provide a path for calculating standard viscoelastic responses from the extracted material characteristics. The new technique based on the Z-transform is complementary to previous model-based viscoelastic analyses and can be advantageous with respect to Fourier techniques due to its generality. Additionally, it can handle the unbounded inputs traditionally used to acquire force–distance relationships in AFM, such as ramp functions, in which the cantilever position is displaced linearly with time for a finite period of time.

## Introduction

Atomic force microscopy (AFM) is a prominent technique for investigating material properties at the micro- and the nanoscale [1–3], within which a wide variety of instruments, probes, and analysis techniques have been developed to attempt meaningful material property extraction [4–11]. With regards to viscoelasticity, efforts that incorporate classical viscoelastic theory [12–16] rely on force–distance curves [17–26], which describe the dependence of the probe–sample interaction force with respect to the probe–surface distance for a particular location on the sample [27]. Force–distance analysis provides direct information on the force and indentation history with respect to time, which makes it appropriate for viscoelastic material property inversion. Existing inversion methods are based on viscoelastic models, which have been developed to describe specific kinds of relaxation behavior in the material. For example, in some cases, the analysis involves an assumption regarding discrete characteristic relaxation timescales in the material, which are represented using spring–dashpot models [20,24], while in other cases a continuous distribution of characteristic times is assumed via power-law rheology models [17,22]. Regardless of the model chosen, the strategy encompasses fitting the properties implied by the model to the experimental force–indentation data.

**Figure 1:**
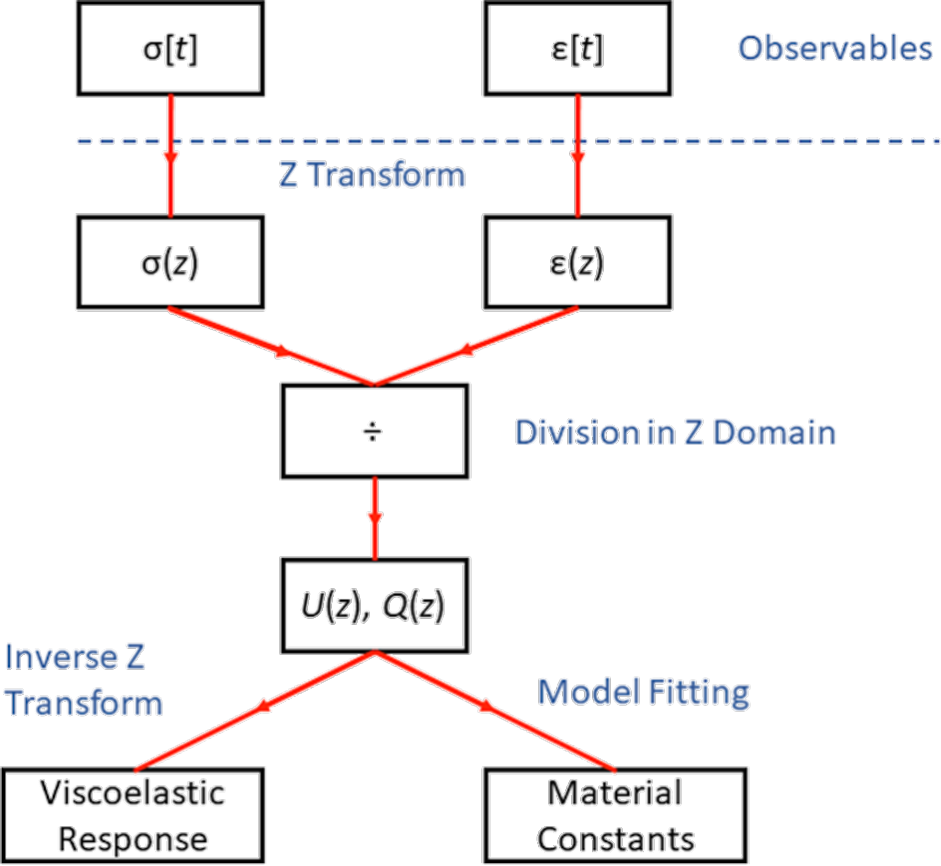
Proposed methodology for viscoelastic analysis utilizing the Z-transform. The stress and strain information is first transformed into the *z*-domain, where ratios of these two variables directly yield the relaxance and retardance. The transfer functions can be used to estimate the time response of the material in the time domain, in numerical form, for specific excitations, using the inverse Z-transform. Alternatively, the transfer functions can be fitted to specific viscoelastic models in order to obtain material constants within those models.

In order to enable a new route to viscoelastic material property inversion, which is complementary to previous strategies, here we propose a paradigmatically different approach that is not based on the choice of a model. Instead of fitting force–distance data to specific functions dictated by approximate models, we transform the experimental information using the Z-transform mathematical technique into the so-called *z*-domain, which is analogous to the Laplace domain, but applicable to discrete finite signals [28]. This enables the extraction of the viscoelastic transfer functions of the material, bypassing the need for any viscoelastic model assumptions (see Figure 1). These transfer functions are the viscoelastic relaxance and retardance, where the relaxance describes the time response of a viscoelastic material to a unit impulse excitation (Dirac delta function) of strain and the retardance describes the response of the material to an impulsive excitation of stress.

**Figure 2:**
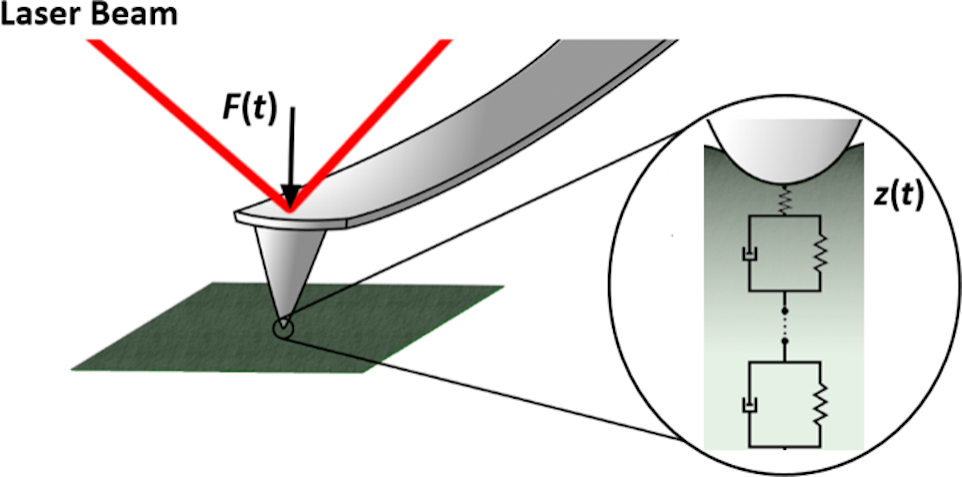
Illustration of the classical force–distance experiment. The AFM cantilever tip approaches and indents the sample under off-resonance conditions, while the force and indentation are calculated with respect to time with the aid of the probe position detection system (e.g., a laser beam detector). The most important assumptions in our linear viscoelastic analysis, which is described in subsequent sections, are that the sample is homogeneous and isotropic, and that the indentation is “small” with respect to the radius of curvature of the indenter. Large sample deformations lead to nonlinear material responses. Further experimental details are provided in the Methods section.

Our proposed model-free approach follows the spirit of related Fourier-based methods, where viscoelastic material extraction has been achieved under some limited circumstances [29–31]. However, in this work, we exploit the advantages of the Z-transform, which we believe is more appropriate for the analysis of viscoelasticity. Guided by the same motivation that has led the classical rheology field to rely on the more general Laplace transform technique for analytical treatments, we rely on the Z-transform to accomplish viscoelastic extraction for finite discrete experimental data. The selection of the Z-transform over the more widely exploited discrete Fourier transform (DFT) will become evident in later sections of the manuscript, where it is shown that our approach has enough generality to obtain meaningful material property information in a model-free fashion, without the periodicity and convergence constraints associated with the DFT, which is better suited for harmonic and/or steady-state excitations. Although the proposed technique can be utilized to analyze different types of experiments, in this work we demonstrate it using classical force–distance experiments (see Figure 2), for which we carry out the force analysis in the complex domain (force spectroscopy).

## Theoretical Background

It is well known that the behavior of viscoelastic materials is history-dependent, as a result of which, the stress–strain relationships governing their deformation are functionals (not functions). More specifically, the stress at a given instant depends on the total previous history of strain and vice versa [13]. This history dependence is often expressed in the form of convolution integrals:

[1] $$\sigma \left(t\right)=\int_{0}^{t}Q\left(t-u\right)\varepsilon \left(u\right)\text{d}u,$$

[2] $$\varepsilon \left(t\right)=\int_{0}^{t}U\left(t-u\right)\sigma \left(u\right)\text{d}u,$$

where *Q*(*t*) and *U*(*t*) are known as relaxance and retardance, respectively. As already stated, relaxance and retardance describe the time response of a viscoelastic material to a unit impulse excitation (Dirac delta function) of either strain or stress, respectively [13]. Theoretically, knowledge of *Q*(*t*) or *U*(*t*) fully characterizes the viscoelastic behavior of the material, so we refer to them as “source” functions.

Linear viscoelasticity exploits the use of the Laplace transform, whose advantage becomes evident upon transforming the previous equations into the Laplace domain and observing the simplicity afforded by this treatment. It is a well-known property of the Laplace transform that convolutions in the time domain, such as the right-hand side of Equation 1 and Equation 2, transform into simple multiplications in the Laplace domain (*s*-domain) [32–33]. Thus, Equations 1 and 2 transform as follows:

[3] σ(s)=Q(s)ε(s),

[4] ε(s)=U(s)σ(s).

From Equation 3 and Equation 4, we can write the relaxance (*Q*(*s*)) and retardance (*U*(*s*)) as operators or transfer functions:

[5] $$Q\left(s\right)=\frac{\sigma \left(s\right)}{\varepsilon \left(s\right)},$$

[6] $$U\left(s\right)=\frac{\varepsilon \left(s\right)}{\sigma \left(s\right)}.$$

From Equation 5 and Equation 6, it is clear that the relaxance is a transfer function with which the stress can be calculated using a given strain input. Likewise, by using the retardance, strain can be calculated for a specific stress input. Clearly also, the relaxance and retardance are inverses of one another in the Laplace domain.

Unit impulse excitations (Dirac delta functions) are mathematically convenient for defining material behavior but are experimentally impractical. Therefore, rheologists normally use a different type of “standard” excitations. The material responses to these standard excitations are known as “standard viscoelastic responses” and are used widely to characterize viscoelastic materials. A very common standard excitation is the harmonic excitation. For example, a harmonic stress input in the time domain that is governed by the angular frequency ω would be represented as:

[7] $$\sigma \left(t\right)=\sigma_{0}e^{i\omega t},$$

which can also be expressed using sine and cosine functions. The corresponding standard viscoelastic response to this harmonic stress excitation in the steady state is calculated by multiplying the appropriate transfer function (the retardance in this case, since the input is stress and the output is strain) by the above stress input. We perform this operation for the case where the Laplace variable *s* equals *i*ω:

[8] $$\varepsilon \left(\omega \right)={\left[U\left(s\right)\right]}_{s=i\omega }\sigma \left(\omega \right)=J^{*}\left(\omega \right)\sigma \left(\omega \right).$$

In the above expression, J*(ω) is the complex compliance, the sinusoidal steady-state strain response to a unit sinusoidal steady-state stress input [12]. From the above equation, it can also be seen that the complex compliance J*(ω) is the Fourier transform of the retardance *U*(*t*). Also, it is a special case of the Laplace-transformed retardance when the complex variable *s* is regarded as purely imaginary:

[9] $${\left[U\left(s\right)\right]}_{s=i\omega }=U\left(i\omega \right)=J^{*}\left(\omega \right).$$

Furthermore, this steady-state complex compliance can be separated into its real and imaginary components:

[10] $$J^{*}\left(\omega \right)=J^{\prime }\left(\omega \right)-iJ^{\prime \prime }\left(\omega \right),$$

where *J*′(ω) and *J*″(ω) are the storage and loss compliance, respectively. The well-known storage and loss moduli can be defined in a similar fashion for the case where a sinusoidal input strain is applied to the material. It is very important to recall the fact that Equations 7–10 and the frequency-dependent quantities included in them refer only to the steady-state case of sinusoidal excitation*.* Most types of AFM characterization do not apply sinusoidal stresses or strains to the material. This is clear with regards to the acquisition of a quasi-static force–distance curve, where the cantilever position above the sample follows a ramp function. In the case of intermittent-contact methods (e.g., tapping-mode AFM), the cantilever tip oscillates nearly sinusoidally, but since tip–sample contact is intermittent, the sample does not experience purely sinusoidal stresses and strains.

To analyze the case of an AFM tip penetrating a viscoelastic surface we need an equation relating force with sample penetration (indentation). Equation 1 and Equation 2 relate stress and strain but do not consider the geometrical aspects of our boundary value problem. Invoking the correspondence principle for the case of a parabolic tip indenting a viscoelastic half-space yields the following force–distance relationship for the time domain [14–15]:

[11] $${\left[h\left(t\right)\right]}^{3/2}=\frac{3}{16\sqrt{R}}\int_{0}^{t}U\left(t-\zeta \right)F\left(\zeta \right)\text{d}\zeta ,$$

where *h* is the sample indentation, *F* is the probe–sample force, and *R* is the radius of the indenter. Transforming this expression into the Laplace domain we obtain:

[12] $$L\left\{h^{3/2},s\right\}=\frac{3}{16\sqrt{R}}U\left(s\right)F\left(s\right).$$

In a force–distance-curve experiment, we have access to both force and indentation history, such that we can, in principle, solve for *U*(*t*). We have previously used this strategy to obtain the time-domain representation of *U*(*t*) within a specific viscoelastic model [19–20]. In the interest of maintaining generality, we have used the generalized Voigt model, for which the retardance has the following form (the generalized Voigt and Maxwell–Wiechert models are discussed in Supporting Information File 1):

[13] $$U\left(t\right)=J_{g}\delta \left(t\right)+\sum_{n}\frac{J_{n}}{\tau_{n}}e^{-t/\tau_{n}},$$

where δ(*t*) is the Dirac delta function, *J*_g_ is the glassy compliance, and *J*_n_ and τ_n_ are the compliance and retardation time, respectively, of the *n*-th Voigt unit in the generalized Voigt model. Physically, these retardation times are the characteristic times at which the molecular rearrangements occur within the viscoelastic material. Replacing the above expression in the time convolution of Equation 11 and simplifying, we obtain the equation we have previously used as the basis for extracting the viscoelastic model parameters and corresponding properties using non-linear least-squares optimization in our previous work:

[14] $$\frac{16\sqrt{R}}{3}{\left[h\left(t\right)\right]}^{3/2}=J_{\text{g}}F\left(t\right)+\sum_{n}\int_{0}^{t}F\left(\zeta \right)\frac{J_{n}}{\tau_{n}}e^{-(t-\zeta )/\tau_{n}}\text{d}\zeta .$$

An alternate strategy is to turn to the Laplace domain equation, working with the special case when *s* = *i*ω, such that the equations are handled more conveniently in Fourier space instead of Laplace space. This seems convenient because, having force and indentation time history, one could envision transforming both of them into Fourier space [29] to obtain the complex compliance. Specifically, letting *s* = *i*ω in Equation 12 and rearranging we obtain:

[15]
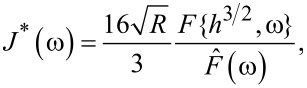


where J*(ω) is the Fourier transform of the retardance, also sometimes denoted as 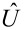
(ω). The convenience is also apparent as many computer packages can efficiently compute the Fourier transform of discrete (experimental) data through the efficient FFT algorithm [34]. However, as we have already pointed out, this only defines the steady-state harmonic response of the material and is therefore not applicable to transient or non-steady-state applications, such as a quasi-static force–distance-curve experiment. This approach is well suited for harmonic excitations but is inappropriate for various types of common experimental inputs such as step excitations or ramp excitations, which is why the more general Laplace transform is widely used in rheology. Methodologies have been developed to circumvent the above restriction of the Fourier transform by applying time derivatives to the experimental quantities and making use of its well-known mathematical properties [29], but although the approaches are theoretically feasible, the existence of noise in AFM signals generally precludes their successful experimental implementation.

## Z-Transform Approach

**Figure 3:**
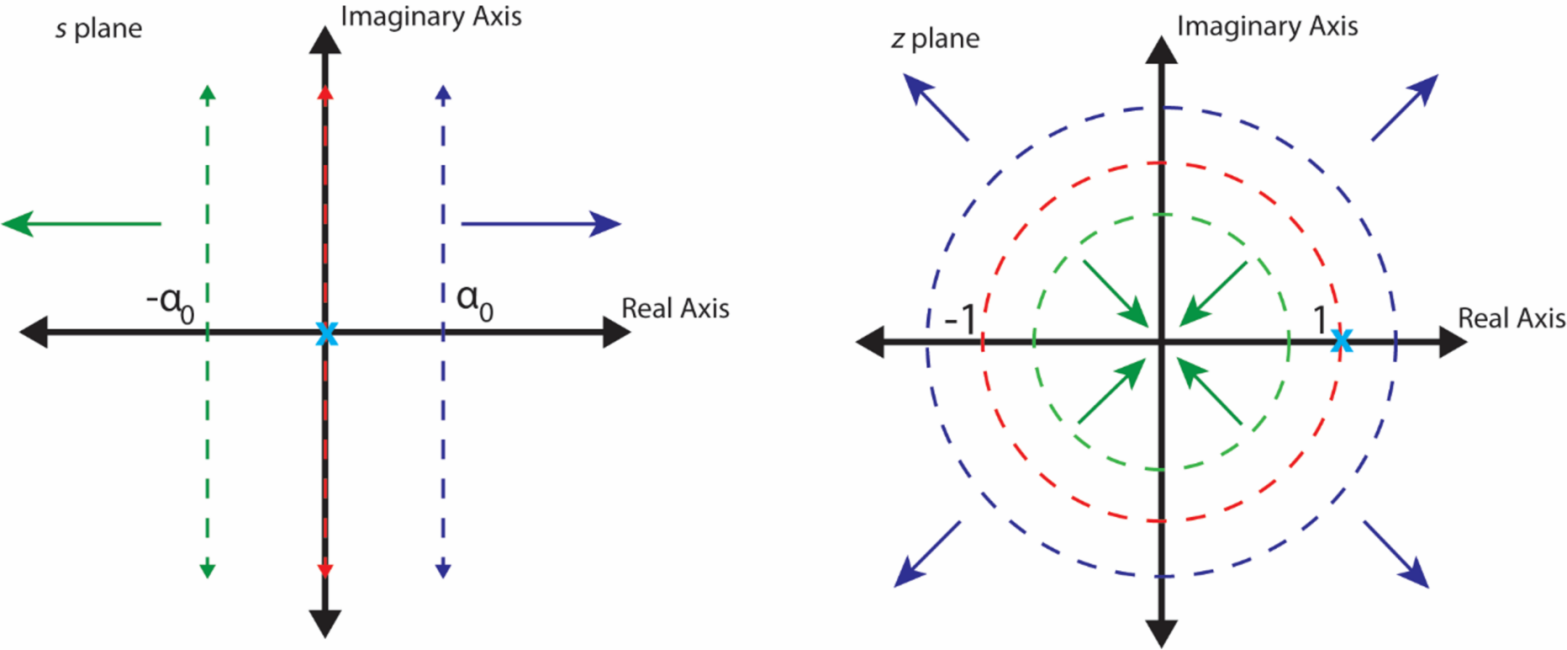
Comparison of the *s*-plane and the *z*-plane. Vertical lines on the *s*-plane map to circles on the z-plane. The imaginary axis on the *s*-plane, which corresponds to the Fourier transform of the signal, corresponds to the unit circle on the *z*-plane. Other lines parallel to the imaginary axis on the *s*-plane correspond to non-unit circles on the *z*-plane. Circles on the *z*-plane, other than the unit circle, correspond to discrete modified Fourier transforms. The *z*-domain can be thought of as an infinite collection of modified Fourier transform domains, along with the Fourier transform domain (unit circle), stitched together.

In this paper, we exploit the wider generality of the Laplace transform to deliver a model-free method to extract the viscoelastic retardance and relaxance of the material from AFM force curves. We focus on the Z-transform, which can be regarded as the discrete-time counterpart to the Laplace transform, and which is, therefore, more general than the discrete-time Fourier transform (a more detailed description of these integral transforms can be found in Supporting Information File 1). In the Laplace transform, a function is mapped into the complex domain via the variable *s* = α + *i*ω, whereas in the Z-transform the function is mapped into the complex domain via the variable *z* = *e*^α^*e^i^*^ω^ (note that the real part of the complex variable *s* is customarily denoted by σ, but here we represent it with α to prevent any confusion with the stress, which is also denoted by σ). Figure 3 provides a comparison between the *s*-plane of the Laplace transform and the *z*-plane of the Z-transform. Based on the relationship between the variables *s* and *z* and disregarding (for now) the fact that the Z-transform is a discrete transform, we see that the *z*-plane is a polar version of the *s*-plane, whereby circles on the *z*-plane correspond to vertical lines on the *s*-plane. For example, the imaginary axis of the *s*-plane, which corresponds to the Fourier transform (*s* = *i*ω, with α = 0), maps to the unit circle on the *z*-plane. It is especially easy to relate the unit circle of the *z*-plane to the discrete Fourier transform. On the unit circle, the angular position of a point can be directly assigned to a frequency value in the discrete Fourier transform. The arc between 0° and −180° (from *z* = 1 to *z* = −1, clockwise) corresponds to frequencies between 0 Hz and the Nyquist frequency (half of the sampling frequency). The complementary arc between 0° and +180° (from *z* = 1 to *z* = −1, counterclockwise) corresponds to frequencies between 0 Hz and the negative of the Nyquist frequency. Other vertical lines on the *s*-plane correspond to non-unit circles on the *z*-plane.

The use of the Z-transform reflects the fact that the experimental data obtained from an AFM experiment always consist of discrete signals. Furthermore, in quasi-static force–distance experiments, the deformation imposed on the material consists of a non-periodic, non-steady-state excitation (e.g., a ramp function). Therefore, the most appropriate transform is one that is discrete and reflects the capabilities of the Laplace transform [35–37].

Reflecting the fact that the stress and strain now consist of the discrete signals σ[*n*] and ε[*n*], respectively, we can write discrete relationships that are analogous to the convolutions presented in Equation 1 and Equation 2:

[16] $$\sigma \left[n\right]=Q*\varepsilon =\sum_{m=0}^{N-1}Q\left[n-m\right]\varepsilon \left[m\right],$$

[17] $$\varepsilon \left[n\right]=U*\sigma =\sum_{m=0}^{N-1}U\left[n-m\right]\sigma \left[m\right],$$

where *N* corresponds to the number of points in the signal. As previously done for writing Equation 3 and Equation 4, we make use of the convolution properties of the Z-transform, which are similar to those of the Laplace transform, to obtain:

[18] σ(z)=Q(z)ε(z),

[19] ε(z)=U(z)σ(z),

where all of the above variables are transformed variables in the *z*-plane. We now write *Q*(*z*) and *U*(*z*) as operators or transfer functions as:

[20] $$Q\left(z\right)=\frac{\sigma \left(z\right)}{\varepsilon \left(z\right)},$$

[21] $$U\left(z\right)=\frac{\varepsilon \left(z\right)}{\sigma \left(z\right)}.$$

We see that the relaxance is a transfer function in the *z*-plane with which stress can be calculated for a given strain input. Likewise, by using the retardance, strain can be calculated for a given stress input. The Z-transforms of the observables are calculated using the definition of the Z-transform:

[22] $$Z\left\{f\left[n\right]\right\}=F\left(z\right)=\sum_{n}f\left[n\right]z^{-n}.$$

Following Equation 12, which reflects the geometry of a spherical indentation experiment, we can write:

[23] $$U\left(z\right)=\frac{16\sqrt{R}}{3}\frac{Z\left\{h^{3/2}\right\}}{F\left(z\right)},$$

[24] $$Q\left(z\right)=\frac{3}{16\sqrt{R}}\frac{F\left(z\right)}{Z\left\{h^{3/2}\right\}}.$$

Similar to our previous methods, these expressions allow for the calculation of the desired transfer functions from experimental data.

## Results and Discussion

### Calculation of the source functions in the *z*-domain

**Figure 4:**
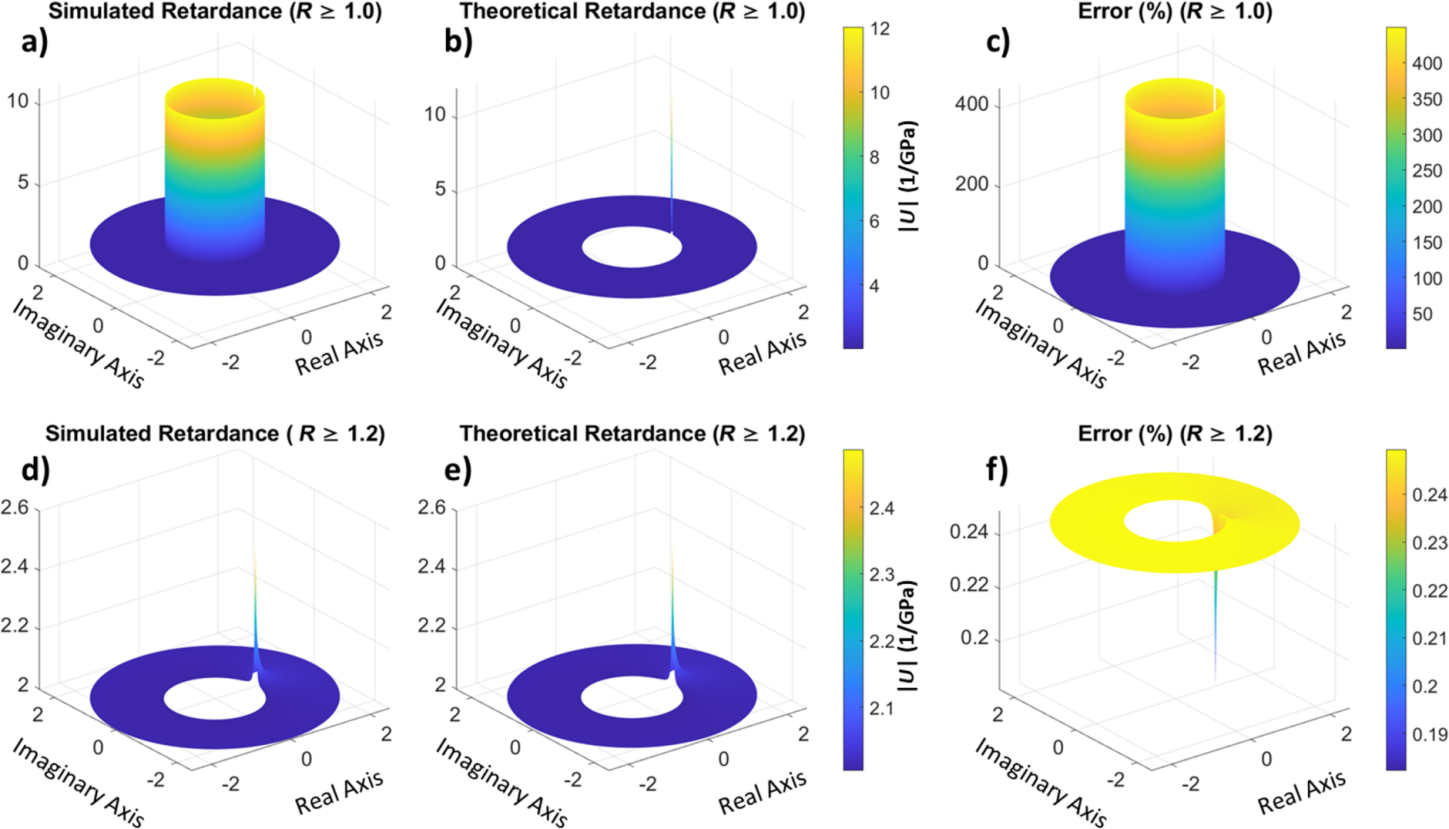
(a, d) Amplitude of the simulated retardance in the *z*-domain for a stress-strain experiment (plot d does not include the unit circle of the *z*-domain). (b, e) Retardance of the material calculated from the theoretical values (plot e does not include the unit circle of the *z*-domain). (c, f) Error between the simulated and theoretical retardance. The second-row figures contain the same information as the first-row figures, except that the data for *R* values smaller than 1.2 has been removed. A large error is observed around the unit circle, which is expected because the unit circle in the Z-transform represents the discrete Fourier transform and in this example we have used non-bounded, non-harmonic input and output [30]. The agreement between theory and simulation is good outside the unit circle (*R* > 1), where every circle represents a modified discrete Fourier transform. The latter corresponds to oscillatory functions multiplied by a decaying (real) exponential function (as in a damped oscillation).

First, to illustrate the proposed technique, we have calculated the retardance of a simulated material. The material is modeled using a generalized Voigt model with a single Voigt unit in series with a spring. Details about the simulation and the material parameters can be found in the Methods section. Linear ramp stress is applied to the material, from which the corresponding strain is calculated. This calculation gives the stress vs strain as a time-dependent array, analogous to an AFM force–distance experiment, which is discussed below. The stress and the strain time series are then transformed into the *z*-domain using Equation 22. Finally, in the *z*-domain, we divide the strain by the stress to obtain the retardance, as prescribed by Equation 21. For comparison, we have also evaluated the retardance from the theoretical equation (Equation 25 in the Methods section) to compare it with our calculated retardance and both are plotted in Figure 4a (calculated) and Figure 4b (theoretical) in the *z*-domain. Apart from the unit circle of the *z*-domain, which corresponds to the Fourier transform of the retardance, the calculated and theoretical retardance are in good agreement (the discrepancy at the unit circle will be discussed below). For *z*-domain points outside of the unit circle, the error between the simulated retardance and the theoretical prediction is less than 0.25%. This is easier to visualize in Figure 4d–f, which do not include the unit circle.

**Figure 5:**
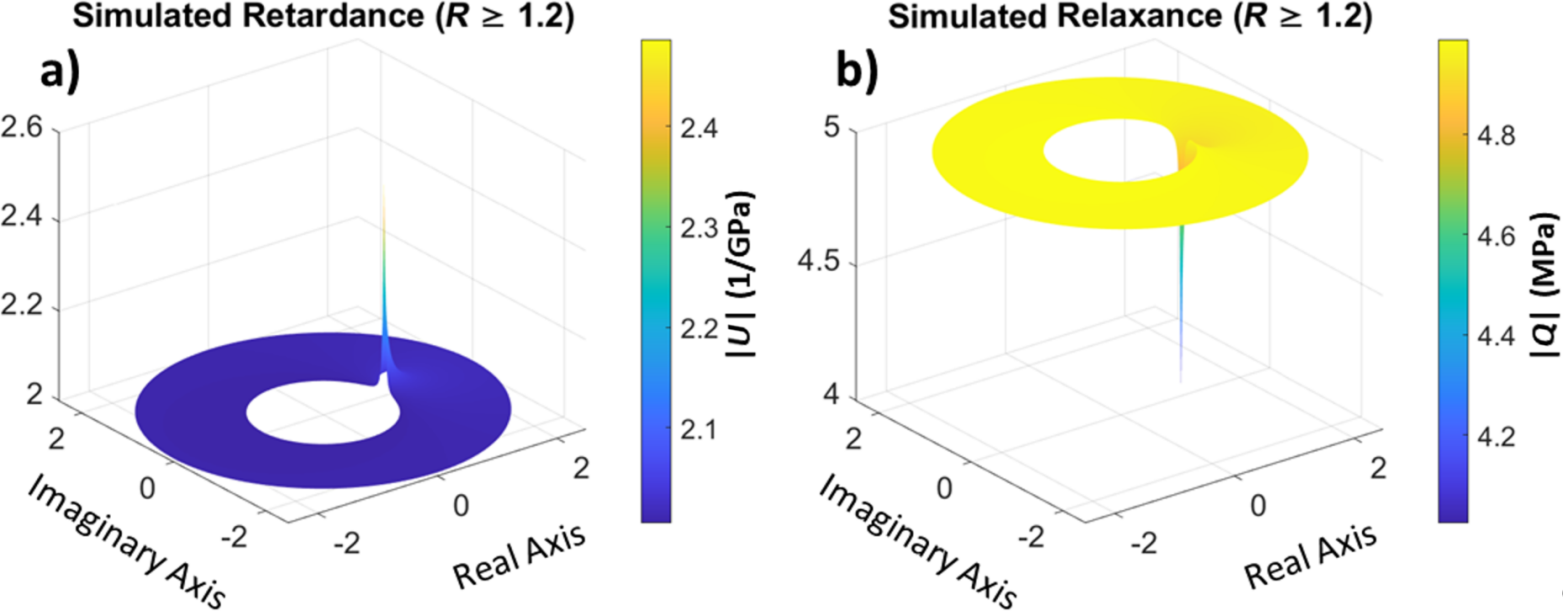
(a) Retardance and (b) relaxance for our simulated material. The retardance is the same as depicted in Figure 4 and the relaxance is directly calculated as its reciprocal.

One of the most significant advantages of working in the complex domain is the ease of inversion of the operators. Unlike the time domain where the inversion of retardance into relaxance and vice versa is challenging and requires either fitting routines for specific models [38–41] or convoluted methods [42], in the *z*-domain, once the retardance is calculated, the relaxance is available through simple inversion of numerator and denominator, as stated in Equation 20 and Equation 21. This inversion is illustrated in Figure 5 for the data provided in Figure 4. Furthermore, although we have used a viscoelastic model to generate the material behavior for our simulation, the calculated retardance and relaxance (Figure 4 and Figure 5) are directly derived from the response of the material and are model-free. Therefore, they do not inherently carry model assumptions or limitations. One may use these material operators in a model-free fashion to predict the material response to an arbitrary stress or strain input, as will be discussed later. Alternatively, it is also possible to fit the calculated retardance (or relaxance) to a viscoelastic model and calculate relevant material constants, characteristic times, and/or loss and storage moduli, as suggested in the bottom-right corner of Figure 1.

### Relationship between the real axis of the *z*-domain and the timescale of the response

**Figure 6:**
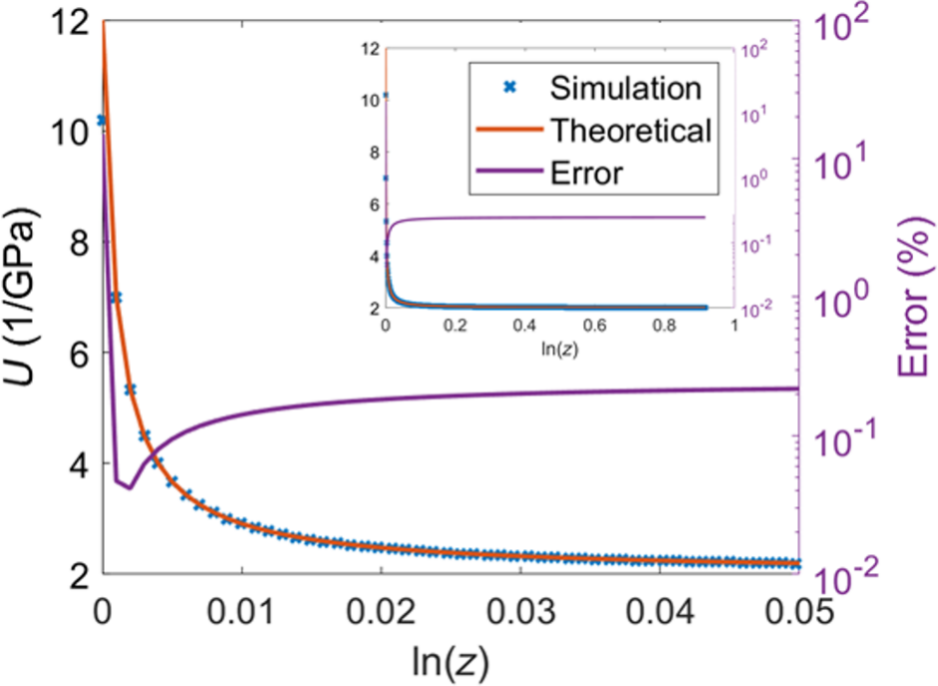
Retardance of the material plotted along the real axis of the *z*-plane using a logarithmic scale. The real axis of the *z*-plane does not contain information about harmonics but instead contains information about the zero-frequency components bounded by exponential functions with different time constants. The point *z* = 1 (this is the vertical-axis intercept on the plot, since the horizontal axis is ln(*z*) and ln(1) = 0), which is located on the unit circle and hence corresponds to the Fourier transform, has a time constant of zero and can be thought of as a representation of the steady-state behavior. As the figure shows, the simulated response at *z* = 1 approaches the equilibrium compliance of the material. The response for large real values of *z* corresponds to faster increasing exponentials, representing the system’s shorter-timescale response. The limit at z→∞ is approached as the sampling time becomes infinitely small.

An interesting feature of the *z*-plane is its real axis. Consider the point *z* = 1, which implies *e*^α^*e^i^*^ω^ = 1, or α = 0 and ω = 0. This point thus corresponds to the zero frequency, or DC component of the Fourier transform. Likewise, other points on this axis correspond to the zero-frequency components of a modified Fourier transform. For example, the point *z* = *b*, where *b* is a real number different than unity, requires ω = 0 (similar to *z* = 1) but corresponds to a non-zero value of α, such that *b* = *e*^α^, where α is positive for *b* > 1 and negative for *b* < 1. If, say, *b* < 1, the point *z* = *b* corresponds to the zero frequency of the modified Fourier transform *e*^−|α|^*e^i^*^ω^, which represents a decaying (damped) oscillation. Thus, depending on their position on the real axis, the points *z* = *b* correspond to either increasing or decreasing exponentials with different time constants, α = ln(*z*). For our purposes, these time constants can be thought of as different timescales for material behavior. For example, at *z* = 1 the time constant is zero, hence our exponential yields a constant steady-state DC component. Increasingly larger *z* values can be thought of as material responses for shorter and shorter timescales. Figure 6 shows the retardance of our example material plotted along the real axis. For this material, the theoretical retardance for *z* = 1 (this is the vertical-axis intercept, since the horizontal axis is ln(*z*) and ln(1) = 0) is the same as the equilibrium compliance of the material, also known as the creep compliance, for which the simulation provides a fair approximation according to Figure 6. As the value of *z* increases along the real axis, the value of the retardance approaches the theoretical glassy compliance, which refers to the material’s infinitely short-timescale response (i.e., instantaneous response), and which is approximated very well by the simulation. We can thus obtain two very important properties of the material quite easily. It is of course not unexpected to obtain the glassy and equilibrium compliances from the real axis, as inspection of Equation 25 (Methods section) shows that those are the expected limits of the retardance:

[26] $$\underset{\left(z,\text{d}t)\to (\infty ,0\right)}{\lim}U=J_{\text{g}},$$

[27] $$U\left(z=0\right)=J_{\text{e}}.$$

### Limitations of Fourier techniques

The Fourier representation of the retardance and the relaxance of the material is commonly used in rheology, where the real components of the operators are referred to as storage modulus or storage compliance, respectively, and the imaginary components as loss modulus or loss compliance, respectively. Although directly obtaining the Fourier representation of the source operators is possible with the proposed method when harmonic or bounded inputs are used, this is not the case for the classical force–distance curve approach, which is based on a ramp function for which the Fourier integral does not converge. Although mathematical tricks can be used for a well-defined function [29,43], additional experimental complications emerge since the signals in an experiment are discrete. When dealing with a bounded function, it can be reasonable to treat the sampling time window as periodic, such that the use of the discrete Fourier transform is warranted. However, this is not appropriate for an unbounded function, which does not possess a finite bandwidth, and for which assuming periodicity leads to signal aliasing. From this consideration we see that the discrete Fourier transform does not necessarily represent the continuous analytical Fourier transform [30], and therefore, a viscoelastic source function calculated using the discrete Fourier transforms of the observables for experiments with unbounded input functions does not correspond to the theoretical harmonic response of the material (Equations 7–10). This is, in fact, the underlying reason for the discrepancy between the theoretical retardance and the calculated retardance in Figure 4 for the unit circle (the Z-transform on the unit circle corresponds to a discrete Fourier transform). In Supporting Information File 1, Figure S6, it is demonstrated that this discrepancy also occurs for the FFT algorithm.

**Figure 7:**
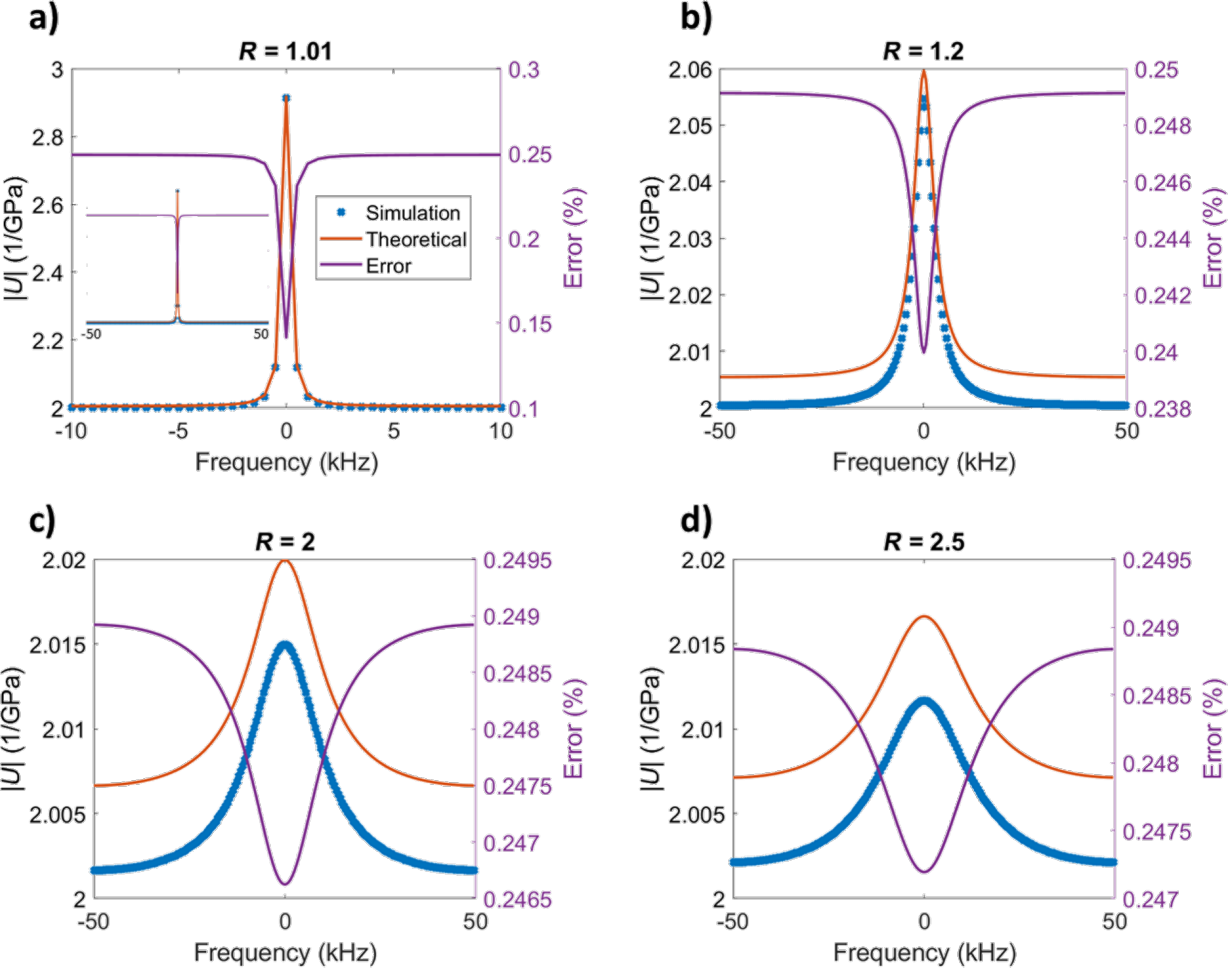
Material retardance amplitude plotted around the origin (i.e., for constant radius and varying frequency – recall that *z* = *e*^α^*e^i^*^ω^), for circles with different radii on the *z*-plane. The radii of the circles are respectively 1.01, 1.2, 2, and 2.5. As the figures show, the retardance calculated from the simulations and the theoretical values match closely with an error below 0.25%. Notice that the smallest error is observed on the real axis in all cases, which corresponds to zero frequency.

Since non-unit circles of the *z*-plane correspond to the modified Fourier transforms of the system, which contain a time-dependent exponential component in addition to their harmonic parts, they can properly represent the characteristics of an experiment with unbounded input. The modified Fourier transforms can properly handle non-steady-state, non-bounded, non-periodic systems, accommodating transient and non-harmonic responses. Figure 7 compares the simulated calculated retardance with the corresponding theoretical values demonstrating good agreement.

**Figure 8:**
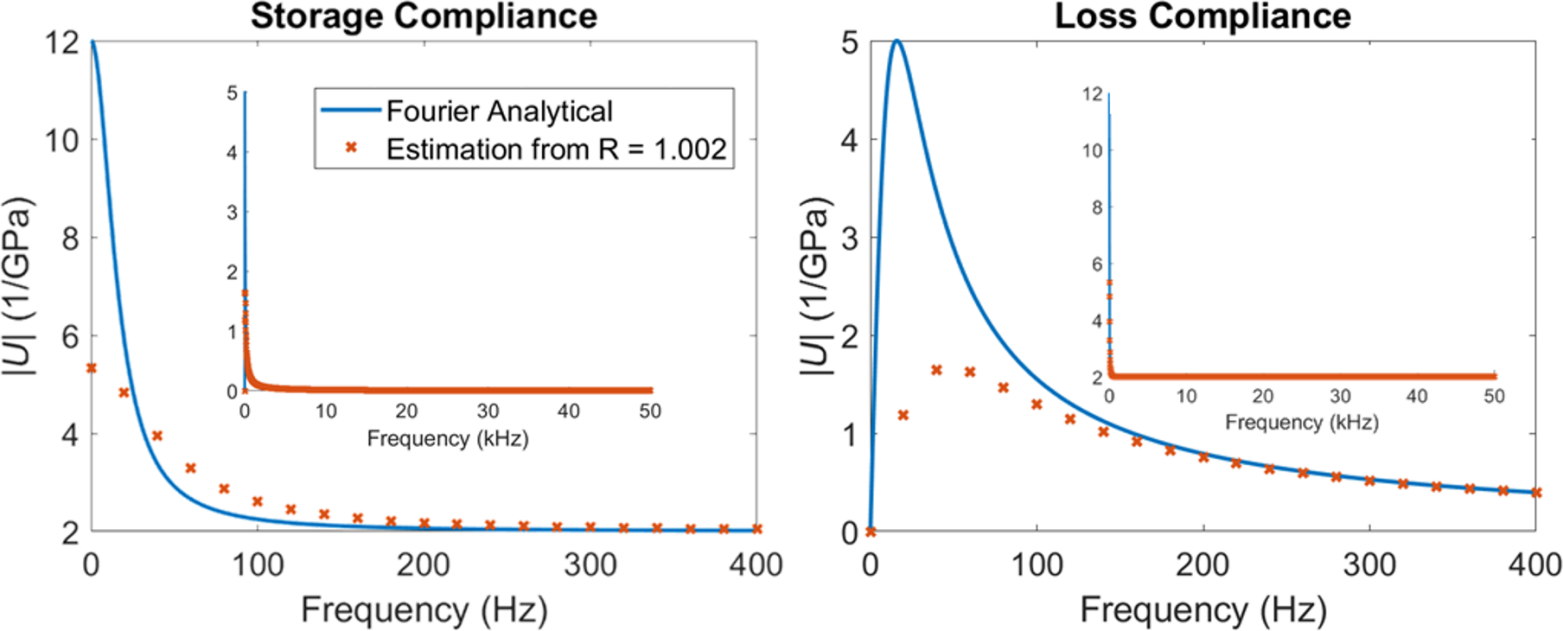
Comparison between the analytical loss and storage compliances for our model material and their estimation from a modified Fourier transform evaluated from the Z-transform. For the generalized Voigt model, the modified Fourier transform versions of the loss and the storage compliances plotted against frequency exhibit peak values that are reduced by a factor of 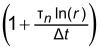 with respect to the Fourier result, while the width of the peaks is increased by a factor of 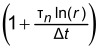. A similar comparison with multiple different modified Fourier transforms can be found in Supporting Information File 1, Figure S9.

As already stated, the storage and loss compliance are not directly accessible from the force–distance experiments, for which the input is unbounded (see also Supporting Information File 1, Figure S6). However, it may be possible to approximate them in some cases by inspecting the real and imaginary components of the neighboring modified Fourier transform regions (i.e., non-unit circles on the *z*-plane which are close to the unit circle). An example is shown in Figure 8 for a circle on the *z*-plane with a radius *r* = 1.002. The error between the estimated storage and loss compliances and the analytical result is small for high frequencies and larger for low frequencies, although in all cases it remains within the order of magnitude of the desired quantities. A much more detailed mathematical analysis of the differences between the Fourier and modified Fourier loss and storage compliances is provided in the Supporting Information File 1. For the generalized Voigt model, the modified Fourier transform version (non-zero time constant) of the magnitudes of the storage and the loss compliance plotted against frequency are reduced by a factor of 
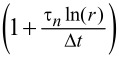
 with respect to the Fourier result (zero time constant). Similarly, the width of the peaks is increased by a factor of 
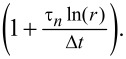
 with respect to the Fourier result and we can observe a compliance behavior that is more spread out over different frequencies.

As illustrated in Figure 8, the loss and storage compliance and their estimation from the modified Fourier transform converge for high frequencies. Therefore, it is possible to accurately estimate the glassy compliance of a material and its immediate response from a force–distance AFM experiment. Furthermore, the position of the local maxima of the loss compliance with respect to frequency changes slightly between the Fourier transform and the modified Fourier transform for the generalized Voigt and Maxwell–Wiechert models, and is given by 
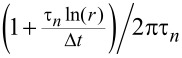
 Hz (a mathematical derivation and a discussion are provided in chapter 6 of Supporting Information File 1). Therefore, the characteristic times can be accurately pinpointed from the modified Fourier transform estimation. Once the characteristic times of the material are estimated, the loss and storage compliances can be calculated from the modified Fourier transforms in light of the peak geometry correction factor discussed above, 
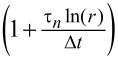
. For this calculation Δ*t* is a known experimental parameter, and *r* is chosen by the researcher.

### Demonstration with AFM contact mechanics

**Figure 9:**
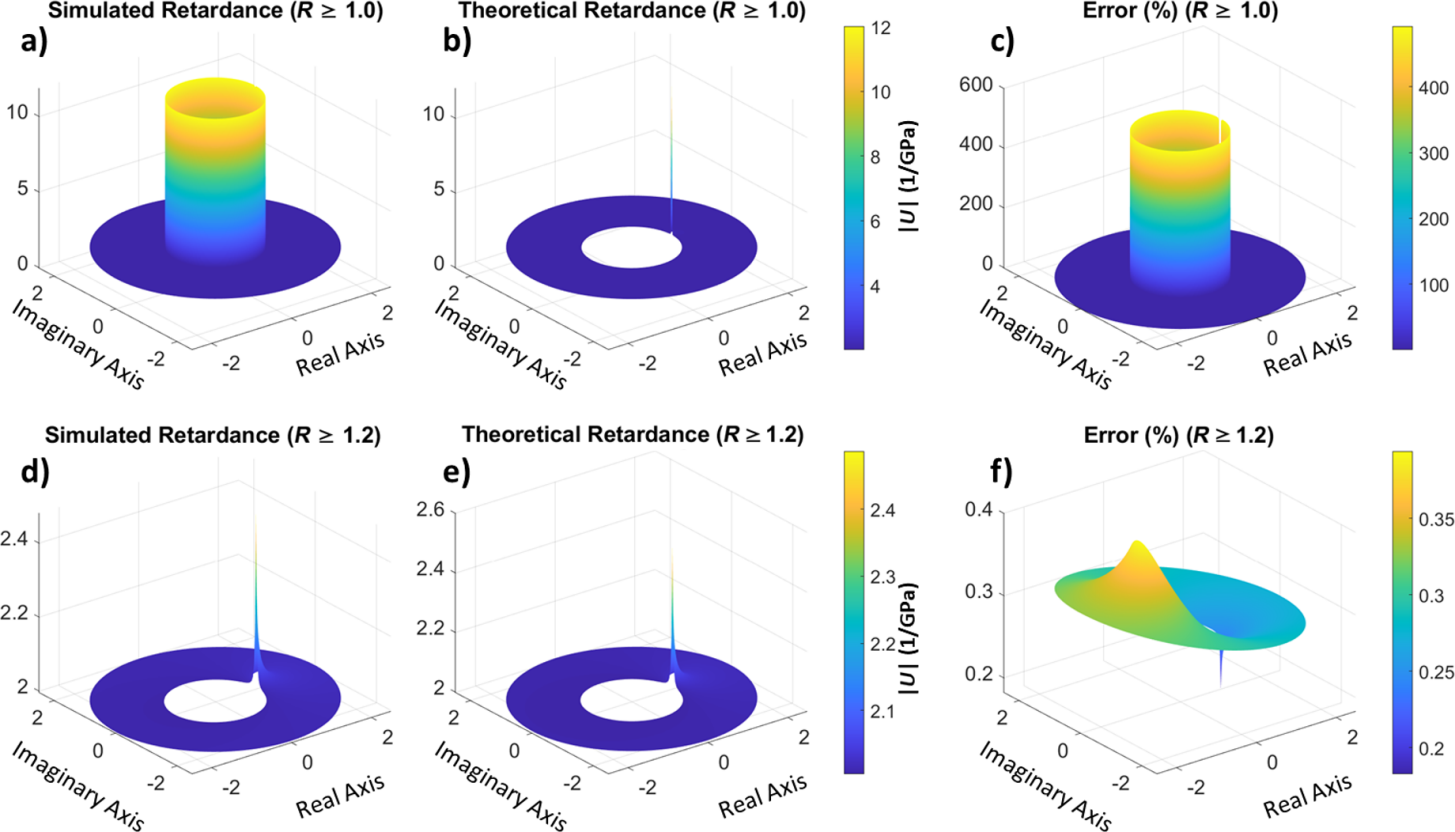
(a, d) Amplitude of simulated retardance in the *z*-domain for an AFM simulation. (b, e) Retardance of the material calculated from the theoretical values. (c, f) Error between the simulation and the theoretical calculations. The information presented in the figures in the top and bottom rows is the same, except that for the bottom row the images only include data for circles with a radius greater than 1.2, while the top row starts from *R* = 1.0. We observe good agreement and similar issues at the unit circle as in Figure 4.

So far, we have demonstrated our method for stress–strain inputs using the generalized Voigt model. However, in AFM experiments one observes the deflection of the AFM cantilever as a function of the cantilever base position instead of directly observing stress and strain. From the available observables, one can calculate the tip–sample force and the indentation. In order to account for the AFM probe and sample geometry and the nature of the corresponding observables, it is necessary to invoke the correspondence principle, through which Equation 11 and Equation 24 are derived. We have simulated an AFM experiment (see Methods section for details), calculating the retardance and comparing it with the theoretical behavior, as shown in Figure 9. As before, we observe a good agreement between simulation and theory, which suggests that the method is also suitable for AFM analysis. Retardance profiles for different circles of the *z*-plane and along the real axis, similar to the data of Figure 6 and Figure 7, can be found in Figures S7 and S8 in Supporting Information File 1.

### Calculation of responses to standard or arbitrary inputs through the inverse Z-transform

**Figure 10:**
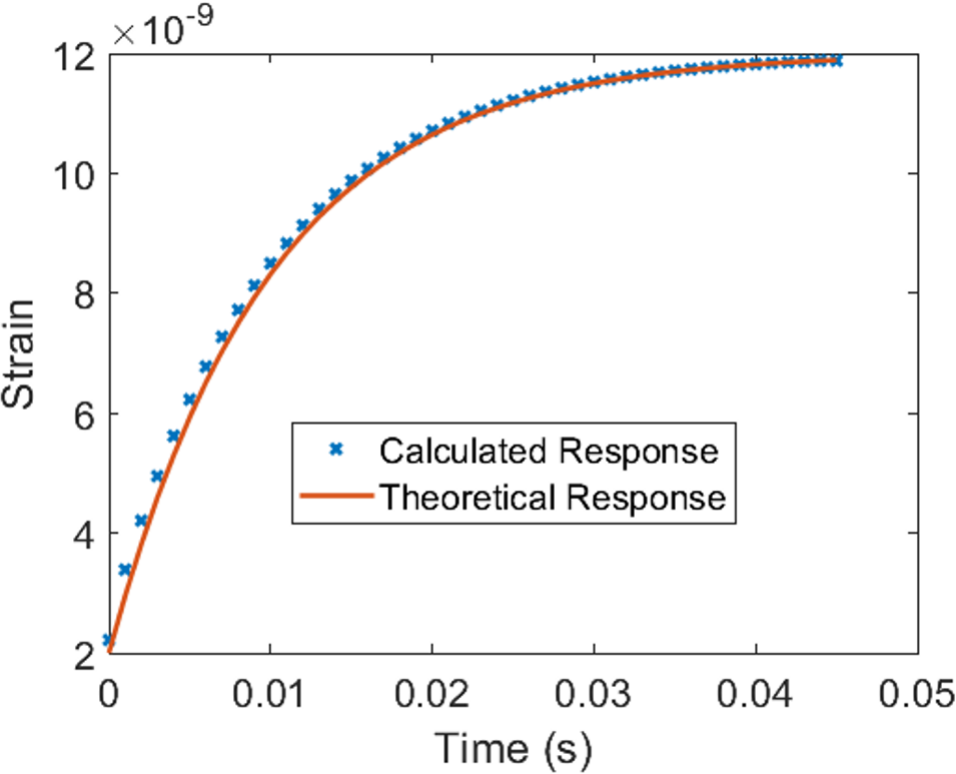
Material response to a step input calculated from the retardance obtained through the previously simulated force–distance experiment (with a ramp force input), and comparison with the theoretical result.

So far we have only discussed the behavior of the retardance and relaxance in the *z*-domain. However, we recall that these operators define the reaction of the material to stress or strain, noting that with the proposed method the representation is model-free. The next logical question is what to do with these operators. Naturally, one option is to fit an established viscoelastic model to them, such as the generalized Voigt or Maxwell–Wiechert models, or any other suitable model. As long as there is a complex domain correspondence between the *z*-plane and the domain of the model, any model can be fit, which yields the parameters of the model. These parameters can then be used to express, for example, compliances, moduli, and characteristic times in analytical form. By extension, one can also obtain the storage and loss moduli. Alternatively, one may continue with a model-free approach, using the operators as they are to predict material responses to specific inputs. For this, all one needs to do is transform the user-defined input into the *z*-domain and multiply with the appropriate operator (calculated relaxance or retardance). The result of this multiplication is the material response in the *z*-domain, from which the time-domain response can be obtained via the inverse Z-transform. Figure 10 shows the result of calculating the creep (strain) response of our model material from the calculated retardance for a unit stress input. The inverse Z-transform is calculated by taking a counterclockwise closed integral along a contour in the *z*-plane (see Equation 28 in the Methods section) [35]. In this work, we have calculated the integral numerically around a non-unit circle, counterclockwise, after evaluating the retardance in the *z*-plane, for convenience.

### Discussion and future work

So far the proposed method seems to be quite promising, but there is one very important consideration that needs to be highlighted, namely computer precision in the calculations. Specifically, modern computational coding languages use variable precision. For example, for a number that is of order unity, the computational precision is ≈10^−16^, but for a number that is of order 10^100^ the precision is ≈10^84^. Therefore, in calculations with large numbers, one can accumulate a massive amount of error, and there are often situations where positive and negative numbers that should cancel each other do not do so due to lack of precision. Unfortunately, this issue plagues the Z-transform due to its numerical nature. While calculating the Z-transform values on the *z*-plane, one calculates the *z*-value’s power −*n*, where *n* scales with the number of sample points in the signal. Likewise, while calculating the inverse Z-transform, one raises *z* to the *n*-th power. During the calculation of the Z-transform, numbers raised to very large negative powers converge to zero, which although not catastrophic, does result in loss of accuracy. On the other hand, while calculating the inverse Z-transform, calculating very large powers can inaccurately diverge to infinity. This can place practical limits on the sample size (number of points in the signal) and may also require very mindful coding.

There are also areas of future work concerning the methods presented here. We have focused on the non-unit circles of the *z*-plane, corresponding to modified Fourier transforms of the operators, where each circle represents a combination of a harmonic response with a time-dependent exponential coefficient having a specific time constant. As previously stated, these exponential coefficients represent non-steady-state behaviors and are crucial to our method. One area of further investigation concerns the relationship between the time constants (or *z*-domain radii) and the time scale of the experiment. Since these time-dependent coefficients lay along the real axis of the *z*-domain (without any harmonic component), the real axis of the *z*-domain contains very important information concerning non-harmonic and non-steady-state behavior in such cases. Non-unit circles with different radii (and hence different time constants) may be more or less important for different operations with different time scales. Furthermore, the non-unit circles of the *z*-domain also contain information about the harmonic behavior of the material (although under damped conditions). Thus, the storage and loss of energy by the material and its relationship to the modified harmonic components should also be investigated further.

**Figure 11:**
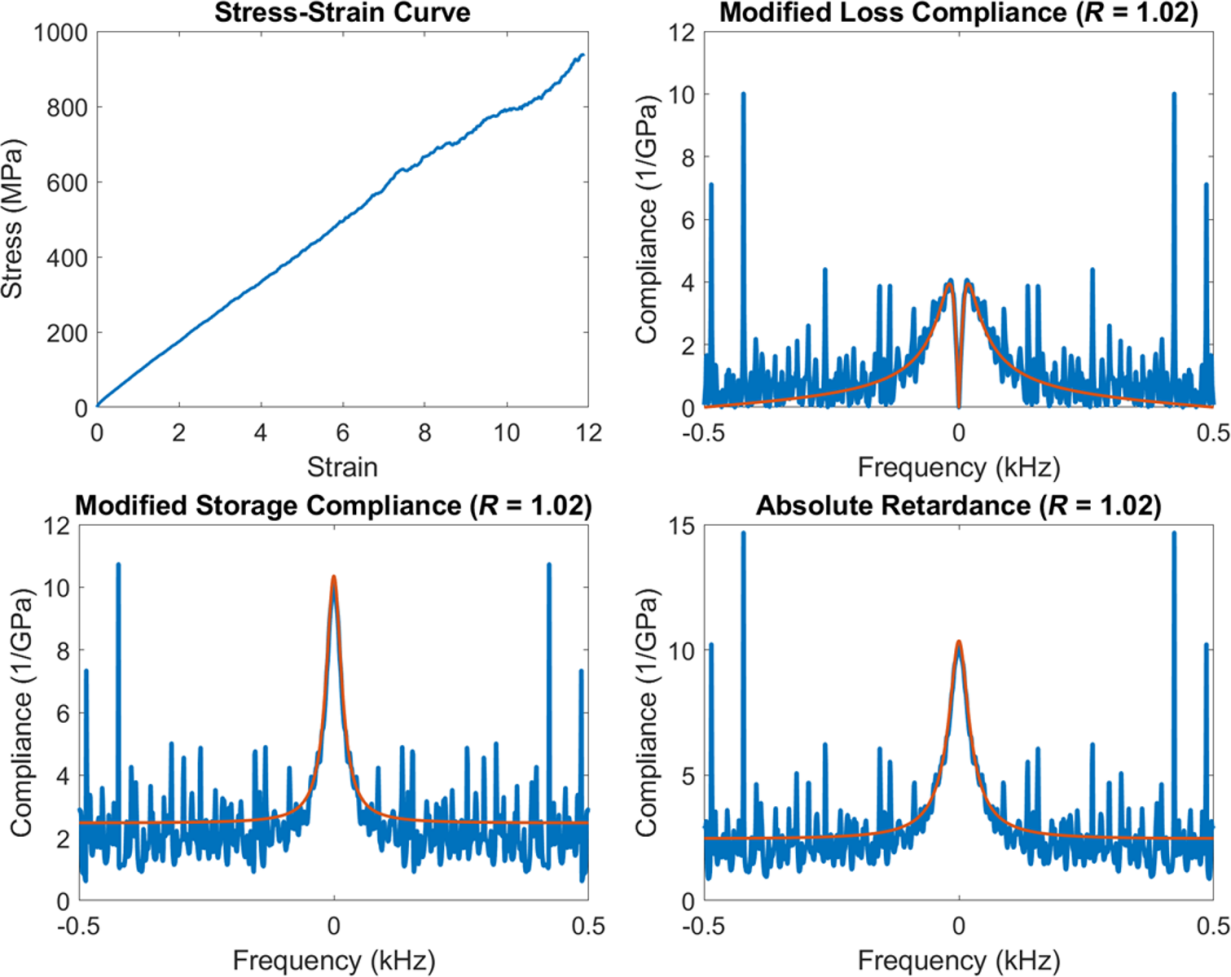
Illustration of the effect of noise in the experimental stress–strain curve on the calculated modified loss compliance, storage compliance and absolute retardance.

One final area of future work concerns the effect of electronic or thermal noise on the quality of the viscoelastic analysis. Generally speaking, analyses in the complex domain can handle noise more robustly than analyses in the time domain, especially when specific features are expected in the plots of the viscoelastic functions. For example, the fact that in the Kelvin–Voigt model, non-zero-centered peaks are not expected in the storage compliance (see Figure 11) could be used to discriminate noise from legitimate material behavior. One can envision the development of smart algorithms that “clean up” the viscoelastic functions based on their expected behavior. However, this is clearly more challenging in our model-free framework, where the features of the material behavior may not be known a priori. Further research is encouraged on this topic.

## Conclusion

A novel method for obtaining the viscoelastic properties of a material using atomic force spectroscopy has been proposed and demonstrated computationally. The method utilizes Z-transform techniques and yields model-free viscoelastic information, such as the material retardance and relaxance, as well as standard viscoelastic responses and information on material behavior at different timescales. The method has advantages over discrete Fourier transform methods, in that it can handle unbounded and non-periodic signals, such as the inputs that are traditionally used in quasi-static AFM force–distance experiments. Furthermore, the acquisition of a transfer function that defines the linear viscoelastic behavior of the material enables the generation of any standard and non-standard viscoelastic material response from it once the input is defined by the user. Although the method provides a way to perform model-free viscoelastic analysis, it is also possible to fit the transfer functions to specific models in order to obtain parametrized analytical expressions for them.

## Methods

The viscoelasticity of the material is simulated by using a single Voigt unit plus a residual spring within the generalized Voigt model (see Supporting Information File 1, Figure S2, Equation S42), which contains one characteristic retardation time τ. The material properties for this model are provided in Table 1.

**Table 1:** Material parameters used in the simulations.

*J*_g_ (Pa)	*J* (Pa)	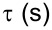

2 × 10^−9^	1 × 10^−8^	0.01

To calculate the material retardance and relaxance in the *z*-domain, a ramp input stress is first defined (Equation 29) and the corresponding strain response is then calculated using Equation 30 below, which gives the theoretical response for the chosen model [13]. The duration of the simulated experiment is 0.1 s and the data is discretized using a timestep of 0.00001 s:

[29] $$\sigma \left(t\right)=10^{9}t,$$

[30] $$\varepsilon \left(t\right)=\left(Jt+J_{\text{g}}t+J\tau \left(e^{\left(-\frac{t}{\tau }\right)}-1\right)\right)\times 10^{9}.$$

The stress and strain signals are subsequently transformed into the *z*-domain (see Equation S80, Supporting Information File 1). One can visualize the process by considering the signals as a succession of time-shifted delta functions, which can be easily transformed into the *z*-domain. The theoretical retardance is evaluated using Equation 25 (see Supporting Information File 1 for its derivation):

[25] $$U=\frac{\varepsilon }{\sigma }=J_{\text{g}}+\sum_{n}\frac{J_{n}}{1+\frac{\tau_{n}}{\Delta t}\left(1-z^{-1}\right)}.$$

A spherical contact, appropriate for an AFM experiment, was simulated using the same material parameters, with a tip radius of 10 nm, which is common in AFM (See also Figure 2). Analogous to the previous simulation, a ramp indentation with a slope of 1 nm/s was applied to the material for 0.1 s. However, this time we have used Simulink to evaluate Equation 12. The equation was solved using a fourth-order Runge–Kutta solver with an integration timestep of 10^−9^ s, which was downsampled to a timestep of 10^−5^ s to simulate the experimental AFM signal. Note that in the case of an AFM experiment the user does not generally prescribe a variation of indentation over time. Instead, one prescribes the time-dependent displacement of the cantilever base towards and away from the sample while measuring the force (via the deflection). However, the simpler simulation described here is appropriate to evaluate the Z-transform methodology because the data acquired through the AFM experiment needs to be transformed into force vs indentation information before it can be used as input for a viscoelastic inversion analysis [20].

To calculate the creep response, stress and strain were evaluated for a ramp input as in Equation 29 and Equation 30, for a shorter time period with a larger time step. The strain was calculated for 0.045 s with a timestep of 0.001 s. The subsequently calculated retardance was multiplied with a unit step input in the *z*-domain:

[31] ε(z)=H(z)U(z).

The above corresponds to the creep response in the *z*-domain. The unit step input in the *z*-domain can be written as:

[32] $$H\left(z\right)=\frac{z}{z-1}.$$

To calculate the creep response in the time domain, the *z*-domain creep response was obtained by numerically integrating the contour along the circle with *R* = 1.14 counterclockwise. The theoretical creep response was evaluated using Simulink with a fourth-order Runge–Kutta solver using a timestep of 10^−6^ s, taking Equation 6 as the transfer function, in the form of Equation S42, with the parameters from Table 1. As in the previous case, the results were downsampled for demonstration purposes.

The inverse Z-transform of an arbitrary function in the *z*-domain, *X*(*z*), can be calculated using the following contour integral [35]:

[28]
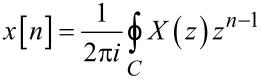


## Supporting Information

The Supporting Information features derivations of generalized Voigt and Maxwell–Wiechert models in the Laplace and the *z*-domains; a brief overview of Fourier, modified Fourier, Laplace, and Z-transforms; additional data illustrating the misrepresentation of the system in the Fourier domain and additional data from AFM simulations; and, finally, a detailed analysis of loss and storage as a function of frequency and their estimation from modified Fourier transforms.

File 1 Additional methodical data.
