# Supplementary material for: A new method for obtaining model-free viscoelastic material properties from atomic force microscopy experiments using discrete integral transform techniques
Source: Beilstein J Nanotechnol. 2021 Sep 23;12:1063–77. doi: 10.3762/bjnano.12.79 (PMC8474069; doi:10.3762/bjnano.12.79)
Supplement: File 1 — Additional methodical data. [file Beilstein_J_Nanotechnol-12-1063-s001.pdf]

BEILSTEIN JOURNAL OF NANOTECHNOLOGY

Supporting Information

for

A new method for obtaining model-free viscoelastic material properties from atomic force microscopy experiments using discrete integral transform techniques

Berkin Uluutku, Enrique A. López-Guerra and Santiago D. Solares

Beilstein J. Nanotechnol. **2021,** *12,* 1063–1077. doi:10.3762/bjnano.12.79

Additional methodical data

License and Terms: This is a supporting information file under the terms of the Creative Commons Attribution License (https://creativecommons.org/licenses/by/4.0). Please note that the reuse, redistribution and reproduction in particular requires that the author(s) and source are credited and that individual graphics may be subject to special legal provisions.
The license is subject to the *Beilstein Journal of Nanotechnology* terms and conditions: (https://www.beilstein-journals.org/bjnano/terms)

Table of Contents

1. RHEOLOGICAL MODELS IN LAPLACE (S-) AND Z-DOMAINS **S3**

A. DERIVATION OF THE GENERALIZED MAXWELL-WIECHERT MODEL IN THE LAPLACE AND Z-DOMAINS S3
B. DERIVATION OF THE GENERALIZED VOIGT MODEL IN THE LAPLACE AND Z-DOMAINS S7

2. DEVISING A DERIVATIVE OPERATOR FOR THE Z-TRANSFORM **S12**

3. FOURIER TRANSFORM, LAPLACE TRANSFORM, AND Z-TRANSFORM: AN OVERVIEW AND RELATIONSHIPS . **S13**

A. FOURIER SERIES AND FOURIER TRANSFORM S14
B. MODIFIED FOURIER TRANSFORM S16
C. LAPLACE TRANSFORM S18
D. Z-TRANSFORM S20

4. FOURIER TRANSFORM OF THE INPUT-OUTPUT AND MISREPRESENTATIONS OF THE SYSTEM **S20**

5. DATA FROM CONTACT MECHANICS SIMULATION **S21**

6. ANALYSIS OF LOSS AND STORAGE HARMONIC VISCOELASTIC FUNCTIONS AND THEIR ESTIMATION FROM MODIFIED FOURIER TRANSFORMS **S23**

A. GENERALIZED VOIGT MODEL S24
B. MAXWELL-WIECHERT MODEL S28

1- Rheological Models in Laplace (*s*-) and *z*-Domains

Here we discuss two commonly used rheological models for linear viscoelasticity: the Maxwell-Wiechert Model, commonly referred to as Generalized Maxwell Model, and the Generalized Voigt Model. Within these models we represent viscoelastic systems using combinations of spring and dashpot elements [1–3]. We should also note that these models are conjugate models. This means that they can simulate the same material and result in the same quantitative behavior with an adequate selection of parameters [2,4]. Before describing these two models we will review the spring and dashpot elements.

The spring element itself behaves like a purely elastic linear spring and follows Hooke's law:

$$\sigma = G\epsilon \tag{S1}$$

σ and ϵ, the stress and strain, respectively, are analogs to the force and displacement in the spring equation, while *G* is the modulus of the element and analogous to the spring constant. Although there is a correspondence with the usual harmonic spring equation, the units here are different. σ and *G* have units of stress (rather than units of force and spring constant) and ϵ is unitless (instead of having units of displacement).

On the other hand, the dashpot (damper) element represents Newtonian viscous damping and by itself is purely viscous. Unlike the spring element, the dashpot element does not produce stress proportional to the strain on the element, but rather produces stress proportional to the rate of the strain:

$$\sigma = \eta\dot{\epsilon} \tag{S2}$$

a. Derivation of the Maxwell-Wiechert Model in the Laplace and *z*-Domains

The Maxwell-Wiechert model consists of multiple Maxwell "arms" in parallel, as illustrated in Figure S1. Before calculating the stress-strain behavior of the entire model with *n* number of Maxwell

arms, we will first investigate the strain-stress relationship of a single Maxwell arm. After deriving a relationship between the stress and the strain, we will seek a transfer function between them in the *s*-domain and the *z*-domain. The transfer function we will derive is called relaxance, which provides the stress generated by a known strain.

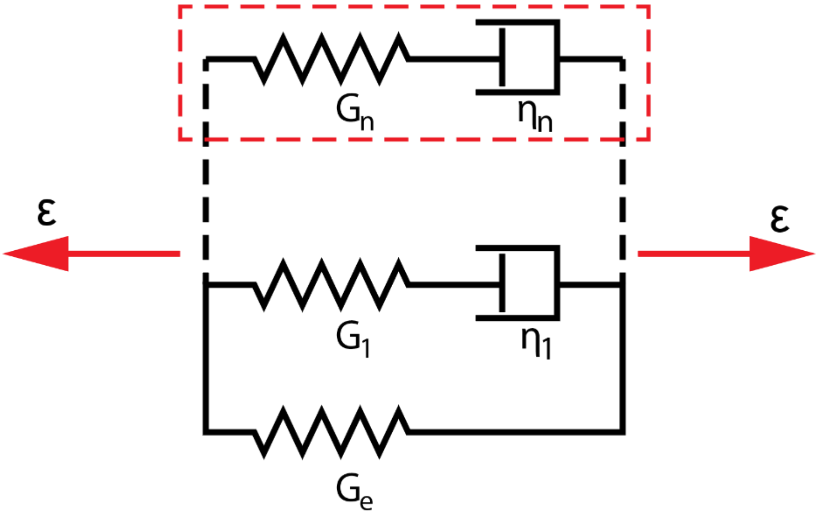

Figure S1: Representation of the Maxwell-Wiechert model. An individual Maxwell element ("arm") is enclosed by the red dashed line. G_n represents the modulus of the spring and η_n represents the viscosity of the dashpot of the nth Maxwell arm.

In a Maxwell arm, a spring element and a dashpot element are arranged in series, and therefore they share the same stress. The total strain on the arm is equal to the summation of the strains of the individual elements:

$$\sigma = \sigma_s = \sigma_d \tag{S3}$$

$$\epsilon = \epsilon_s + \epsilon_d \tag{S4}$$

$$\dot{\epsilon} = \dot{\epsilon}_s + \dot{\epsilon}_d \tag{S5}$$

By combining Equations S1 to S5 we can write a differential relationship between the strain and the stress of the arm:

$$\dot{\epsilon} = \dot{\epsilon}_s + \dot{\epsilon}_d = \frac{\dot{\sigma}}{G} + \frac{\sigma}{\eta} \tag{S6}$$

The ratio of the viscosity and the elasticity gives the "relaxation time" for this arm, which is a commonly used parameter. We can write Equations S5 and S6 in terms of the relaxation time:

$$\tau = \frac{\eta}{G} \tag{S7}$$

$$\dot{\epsilon} = \frac{\dot{\sigma}}{G} + \frac{\sigma}{G\tau} \tag{S8}$$

We now have a differential relationship between the strain and the stress of the arm. This differential relation is quite convenient to deal with in the Laplace domain, where we can easily find a transfer function between the strain and the stress, the relaxance. We can write equation S7 in the Laplace domain as:

$$\epsilon s = \frac{\sigma s}{G} + \frac{\sigma}{G\tau} \tag{S9}$$

$$\epsilon s = \sigma \left(\frac{s}{G} + \frac{1}{G\tau} \right) \tag{S10}$$

$$\epsilon s = \sigma \left(\frac{\tau s + 1}{G\tau} \right) \tag{S11}$$

$$\epsilon = \sigma \left(\frac{\tau s + 1}{G\tau s} \right) \tag{S12}$$

$$\sigma = \epsilon \left(\frac{G\tau s}{\tau s + 1} \right) \tag{S13}$$

The relaxance of a single maxwell arm can therefore be written as:

$$Q = \frac{\sigma}{\epsilon} = \left(\frac{G\tau s}{\tau s + 1} \right) \tag{S14}$$

To find the stress-strain behavior of a Maxwell-Wiechert model we need to consider its individual arms. Since the arms are arranged in parallel, the total stress of the system is going to be equal to the summation of the stress contributions of the individual arms. However, the total strain of the system is going to be the same for all arms. Therefore, we can write the stress-strain relationship of a Maxwell-Wiechert model with a spring in parallel with *n* Maxwell arms using equation S13 as:

$$\sigma = \sum_n \sigma_n = \epsilon\left(G_e + \sum_n \frac{G_n \tau_n s}{\tau_n s + 1}\right) \tag{S15}$$

From here we can find the relaxance of the system as:

$$Q = \frac{\sigma}{\epsilon} = G_e + \sum_n \frac{G_n \tau_n s}{\tau_n s + 1} \tag{S16a}$$

$$Q = \frac{\sigma}{\epsilon} = G_g - \sum_n \frac{G_n}{\tau_n s + 1} \tag{S16b}$$

To derive an expression for the relaxance in the *z*-domain, we will treat our differential equation (Equation S8) very similarly to how we treated it in the Laplace domain. However, we will use our differentiation for *z*-transform, which is discussed in the second section of this document. We will first find the stress-strain relationship of an individual Maxwell arm, and afterwards we will derive an expression for the Maxwell-Wiechert model response and its *relaxance* using the individual arm result.

One crucial item to notice before starting our derivation concerns the units and treatment of time in the *z*-domain. The Z-transform of a signal is calculated according to individual elements of that signal. Therefore the signal is *not* treated as being a function of time, but rather as a function of n, the time step index (we will thus be dealing with time steps rather than with time itself). Therefore, we ought to convert constants of time (such as the characteristic time and the viscosity) to constants of time steps. We will carry out this task by dividing them by the size of the time step, which will prevent unit mismatches. In our analysis we will be mindful of how many time steps have elapsed, rather than how much time has elapsed. Using the derivation method of the next section, we can write for a single Maxwell arm (see Equation S8):

$$\epsilon(1 - z^{-1}) = \frac{\sigma(1 - z^{-1})}{G} + \frac{\sigma}{G\frac{\tau}{\Delta t}} \tag{S17}$$

$$\epsilon(1 - z^{-1}) = \sigma\left(\frac{(1 - z^{-1})}{G} + \frac{1}{G\tau}\right) \tag{S18}$$

$$\epsilon(1 - z^{-1}) = \sigma \left(\frac{\frac{\tau}{\Delta t}\,(1 - z^{-1}) + 1}{G\frac{\tau}{\Delta t}} \right) \tag{S19}$$

$$\epsilon = \sigma \left(\frac{\frac{\tau}{\Delta t}\,(1 - z^{-1}) + 1}{G\frac{\tau}{\Delta t}\,(1 - z^{-1})} \right) \tag{S20}$$

$$\sigma = \epsilon \left(\frac{G\frac{\tau}{\Delta t}}{\frac{\tau}{\Delta t}(1 - z^{-1}) + 1} \right)(1 - z^{-1}) \tag{S21}$$

which leads to the relaxance of a single maxwell arm:

$$Q = \frac{\sigma}{\epsilon} = \left(\frac{G\frac{\tau}{\Delta t}}{\frac{\tau}{\Delta t}(1 - z^{-1}) + 1} \right)(1 - z^{-1}) \tag{S22}$$

Similar to the transition between Equation S14 and Equations S15 and S16, we can find the strain-stress relationship and the relaxance of a Maxwell-Wiechert model as:

$$\sigma = \sum_n \sigma_n = \epsilon \left(G_e + \sum_n \frac{G_n\frac{\tau_n}{\Delta t}}{\frac{\tau_n}{\Delta t}(1 - z^{-1}) + 1}(1 - z^{-1}) \right) \tag{S23}$$

$$Q = \frac{\sigma}{\epsilon} = G_e + \sum_n \frac{G_n\frac{\tau_n}{\Delta t}}{\frac{\tau_n}{\Delta t}(1 - z^{-1}) + 1}(1 - z^{-1}) \tag{S24}$$

b. Derivation of the Generalized-Voigt Model in the Laplace and *z*-Domains

The Generalized Voigt Model consists of multiple Voigt elements as shown in Figure S2. We will carry out a derivation similar to the derivation we have carried out for the Generalized Maxwell-Wiechert model. We will explore the strain-stress relationship of a single Voigt unit, after which we will calculate the stress-strain behavior of the entire model with *n* Voigt units. In contrast to the Generalized Maxwell-Wiechert model, here it is customary to use the compliance to describe the spring elements and the fluidity to describe the dashpot elements, rather than modulus and viscosity.

To keep the parallelism, we will start the derivation using the modulus and viscosity and will introduce the compliance and fluidity at the end. Using compliance and fluidity is also convenient in the Generalized Voigt model due to the serial connection of the Voigt units, additional spring, and additional dashpot. After deriving the relationship between the stress and the strain, we will look for a transfer function between them in the Laplace and z-domains. The desired function is called *retardance,* which gives the strain from a known stress.

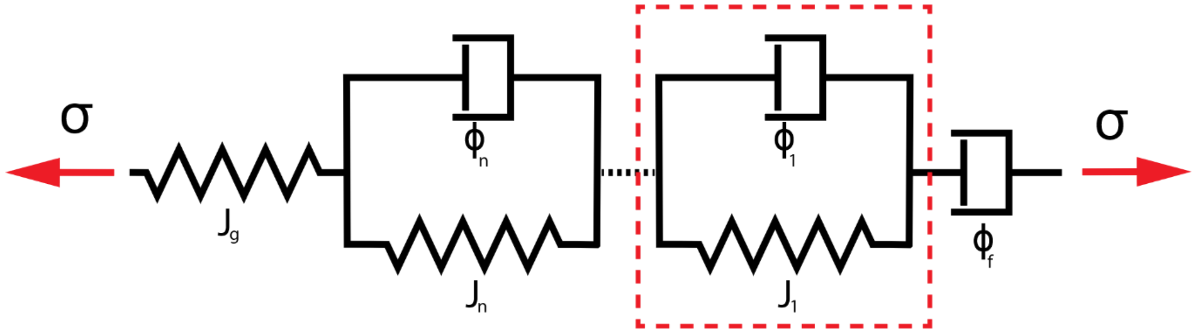

Figure S2: Representation of the Generalized Voigt model. An individual Voigt unit is enclosed by the red dashed line. If the material exhibits steady-state flow behavior, a dashpot of fluidity ϕ_f can be added in series. J_n represents the compliance of the spring and ϕ_n represents the fluidity of the dashpot of the nth Voigt unit.

In a Voigt unit, a spring element and a dashpot element are connected in parallel. This means that, while they undergo the same amount of strain, the total stress of the element is equal to the summation of the stress carried by the individual elements:

$$\sigma = \sigma_s + \sigma_d \tag{S25}$$

$$\epsilon = \epsilon_s = \epsilon_d \tag{S26}$$

By combining equations S1, S2, S25, and S26, we can write a differential relationship between the strain and the stress of the element:

$$\sigma = G\epsilon + \eta\dot{\epsilon} \tag{S27}$$

which can be written in the Laplace domain as:

$$\sigma = G\epsilon + \eta\epsilon s \quad \text{(S28)}$$

$$\sigma = \epsilon(G + \eta s) \quad \text{(S29)}$$

$$\epsilon = \sigma \frac{1}{G + \eta s} \quad \text{(S29)}$$

At this point, we can replace the modulus with the compliance and the viscosity with the fluidity. As their names suggest, the new quantities are inverses of the old ones:

$$J = \frac{1}{G} \quad \text{(S30)}$$

$$\phi = \frac{1}{\eta} \quad \text{(S31)}$$

Therefore, our stress-strain relationship becomes:

$$\epsilon = \sigma \frac{1}{\frac{1}{J} + \frac{1}{\phi} s} \quad \text{(S32)}$$

$$\epsilon = \sigma \frac{J}{1 + \frac{J}{\phi} s} \quad \text{(S33)}$$

By combining the compliance and the fluidity we can define the retardation time of the Voigt unit as:

$$\tau = \frac{J}{\phi} \quad \text{(S34)}$$

With the introduction of the retardation time, we can have a tidier expression:

$$\epsilon = \sigma \frac{J}{1 + \tau s} \quad \text{(S35)}$$

from which we directly obtain the retardance of an individual Voigt element as:

$$U = \frac{\epsilon}{\sigma} = \frac{J}{1 + \tau s} \quad \text{(S36)}$$

To find the stress-strain behavior of a Generalized Voigt model, we need to consider each Voigt unit and the additional spring, as shown in Figure S2. Since the units are arranged in series, the total strain of the system equals the summation of the individual units' strains, while the total stress equals the

stress of an individual unit. We can write the stress-strain relationship of a Generalized Voigt model using equation S35 as:

$$\epsilon = \sum_n \epsilon_n = \sigma\left(J_g + \sum_n \frac{J_n}{\tau_n s + 1}\right) \tag{S37}$$

If the material exhibits steady-state fluidic behavior, we must consider the additional dashpot shown in Figure S2. The strain from that additional dashpot can be calculated from equation S2 and added to the total strain:

$$\sigma = \eta_f \dot{\epsilon} = \phi_f \dot{\epsilon} \tag{S38}$$

$$\sigma = \phi_f \epsilon s \tag{S39}$$

$$\epsilon = \sigma \frac{\phi_f}{s} \tag{S40}$$

$$\epsilon = \sum_n \epsilon_n = \sigma\left(J_g + \sum_n \frac{J_n}{\tau_n s + 1} + \frac{\phi_f}{s}\right) \tag{S41}$$

from which the total retardance of the system is:

$$U = \frac{\epsilon}{\sigma} = J_g + \sum_n \frac{J_n}{\tau_n s + 1} + \frac{\phi_f}{s} \tag{S42}$$

To derive an expression for the retardance in the *z*-domain, we will treat our differential equation (Equation S27) similar to how we treated it in the Laplace domain, using the Z-transform differentiation method discussed in the next section. We will first find the stress-strain relation for an individual Voigt unit, after which we will derive the Generalized Voigt model response and its *retardance*. As for the Maxwell-Wiechert model, we need to convert constants of time to constants of time steps by dividing them by the size of the time step. The stress for an individual unit is:

$$\sigma = G\epsilon + \frac{\eta}{\Delta t}\epsilon(1 - z^{-1}) \tag{S43}$$

$$\sigma = \epsilon\left(G + \frac{\eta}{\Delta t}(1 - z^{-1})\right) \tag{S44}$$

$$\epsilon = \sigma \frac{1}{G + \frac{\eta}{\Delta t}(1 - z^{-1})} \tag{S45}$$

We now replace the modulus with the compliance and the viscosity with the fluidity:

$$J = \frac{1}{G} \tag{S46}$$

$$\phi = \frac{1}{\eta} \tag{S47}$$

From which our stress-strain relationship becomes:

$$\epsilon = \sigma \frac{1}{\frac{1}{J} + \frac{1}{\phi \Delta t}(1 - z^{-1})} \tag{S48}$$

$$\epsilon = \sigma \frac{J}{1 + \frac{J}{\phi \Delta t}(1 - z^{-1})} \tag{S49}$$

Introducing the retardation time we obtain:

$$\epsilon = \sigma \frac{J}{1 + \frac{\tau}{\Delta t}(1 - z^{-1})} \tag{S50}$$

from which we can write the retardance of an individual Voigt element:

$$U = \frac{\epsilon}{\sigma} = \frac{J}{1 + \frac{\tau}{\Delta t}(1 - z^{-1})} + J_g \tag{S51}$$

Using the approach presented above, we can write the strain-stress relationship of a Generalized Voigt model with a spring and *n* Voigt units in series using equation S51 as:

$$\epsilon = \sum_n \epsilon_n = \sigma \left(J_g + \sum_n \frac{J_n}{1 + \frac{\tau}{\Delta t}(1 - z^{-1})} \right) \tag{S52}$$

Including the additional dashpot for the case where the material displays steady-state fluidic behavior:

$$\sigma = \eta_f \dot{\epsilon} = \frac{1}{\phi_f} \dot{\epsilon} \tag{S53}$$

$$\sigma = \frac{1}{\phi_f \Delta t}\epsilon(1 - z^{-1}) \tag{S54}$$

$$\epsilon = \sigma \frac{\phi_f \Delta t}{(1 - z^{-1})} \tag{S55}$$

$$\epsilon = \sum_n \epsilon_n = \sigma\left(J_g + \sum_n \frac{J_n}{1 + \frac{\tau}{\Delta t}(1 - z^{-1})} + \frac{\phi_f \Delta t}{(1 - z^{-1})}\right) \tag{S56}$$

From which the retardance of the system can be written as:

$$U = \frac{\epsilon}{\sigma} = J_g + \sum_n \frac{J_n}{1 + \frac{\tau}{\Delta t}(1 - z^{-1})} + \frac{\phi_f \Delta t}{(1 - z^{-1})} \tag{S57}$$

2- Devising a Derivative Operator for the Z-Transform

In this work we mainly deal with experimental results in the form of numerical, finite and discrete signals. There are numerous ways of approximating differentiations in numerical methods and discrete systems. In this section, we will differentiate in the *z*-domain using the backward difference, whose expression looks very much like the definition of a derivative:

$$\frac{df}{dn} = \frac{f[n] - f[n-1]}{\Delta n = 1} = f[n] - f[n-1] \tag{S58}$$

Here the value of f changes with respect to the index of the element (*n*) and we do not have information about time by itself. However, in cases where we have an accompanying signal which determines the progression of time with *n*, we can define a time derivative using the chain rule:

$$\frac{df}{dt} = \frac{df}{dn}\frac{dn}{dt} = \frac{f[n] - f[n-1]}{\Delta n = 1}\frac{\Delta n = 1}{t[n] - t[n-1]} = \frac{f[n] - f[n-1]}{t[n] - t[n-1]} \tag{S59}$$

The difference between $f[n]$ and $f[n-1]$ is a single element, so we can represent $f[n-1]$ in terms of $f[n]$ in the z domain with a delay [5,6]:

$$\mathcal{Z}\{f[n]\} = F(z) \tag{S60a}$$

$$\mathcal{Z}\{f[n-1]\} = z^{-1}F(z) \tag{S60b}$$

Using this time delay relation, we can re-write Equation S58 as:

$$\mathcal{Z}\left\{\frac{df}{dn}\right\} = \mathcal{Z}\{f[n] - f[n-1]\} = F(z) - z^{-1}F(z) = (1 - z^{-1})F(z) \tag{S61}$$

from which we can write the derivative operator in the *z*-domain as:

$$\mathcal{Z}\left\{\frac{d}{dn}\right\} = 1 - z^{-1} \tag{S62}$$

We can proceed similarly for the second derivative:

$$\mathcal{Z}\left\{\frac{d^2f}{dn^2}\right\} = \mathcal{Z}\{(f[n] - f[n-1]) - (f[n-1] - f[n-2])\} \tag{S63}$$

$$= \mathcal{Z}\{f[n] - 2f[n-1] - f[n-2]\} = (1 - 2z^{-1} + z^{-2})F(z)$$

From here, the operator for the second derivative can be written as:

$$\mathcal{Z}\left\{\frac{d^2}{dn^2}\right\} = 1 - 2z^{-1} + z^{-2} \tag{S64}$$

Generalizing, we can write the mth-order derivative and its respective operator as:

$$\mathcal{Z}\left\{\frac{d^m f[n]}{dn^m}\right\} = (1 - z^{-1})^m F(z) \tag{S65}$$

$$\mathcal{Z}\left\{\frac{d^m}{dn^m}\right\} = (1 - z^{-1})^m \tag{S66}$$

3- Fourier Transform, Laplace Transform, and Z-Transform: An Overview and Relationships

This section provides an overview of the family of the Fourier transform, Laplace transform, and Z-transform. The Fourier transform is a very common mathematical technique that is used in

many different fields. It is arguably the most famous integral transform and it is also relatively easy to visualize for users in multiple fields. Likewise, the Laplace transform is a commonly used technique and a tool for dealing with differential equations in engineering and applied sciences. Although the Z-transform is also well-known and widely used in digital signal processing, it may be unfamiliar and difficult to visualize for users from other fields. In reviewing these transform techniques here we will discuss their similarities and differences in a succinct fashion, aiming at providing the required basic knowledge to users from fields in which they are not used. We also aim to share the insight that led to the approach used in our viscoelastic inversion methods. In writing this section we have used and benefited from very useful reference books [5–8], which we highly recommend to readers who would like to explore the subject more deeply.

a. Fourier Series and Fourier Transform

In the original work of Fourier, where he investigates the heat equation, he claims that any periodic and continuous function can be represented with a summation of properly chosen sine and cosine waves. Although it was a controversial claim back in the early 1800s, in modern-day science and engineering we use *"properly chosen sine and cosine waves"* very frequently. It is important to note that, in order to be able to represent our signal (or function) as a Fourier series, our signal needs to be *continuous and periodic.* We can express the Fourier series for a periodic signal with a period of *T* in different forms to our liking and convenience, where the expressions are mathematically equivalent given appropriately chosen coefficients:

$$f(t) = \frac{a_0}{2} + \sum_n \left(a_n \cos\left(\frac{2\pi nt}{T}\right) + \mathrm{b_n sin}\left(\frac{2\pi nt}{T}\right)\right) \tag{S67}$$

$$f(t) = \frac{A_0}{2} + \sum_n \left(A_n \cos\left(\frac{2\pi nt}{T} + \phi_n\right)\right) \tag{S68}$$

$$f(t) = \sum_n \left(c_n e^{\frac{2\pi i n t}{T}} \right) \tag{S69}$$

By considering a signal that has the period $T \to \infty$, we can derive the definition of the Fourier transform. Importantly, in the Fourier series the frequency difference between consecutive sinusoidal functions is $\frac{2\pi}{T}$. By considering $T \to \infty$, we make this frequency difference infinitesimally small. Skipping intermediate steps we can proceed as follows [7]:

$$\omega = \frac{2\pi n}{T} \tag{S70}$$

$$\Delta\omega = \frac{2\pi}{T} \tag{S71}$$

$$\lim_{T\to\infty} \Delta\omega = d\omega \tag{S72}$$

$$\mathfrak{F}\{f(t)\} = F(\omega) = \int_{-\infty}^{\infty} f(t) e^{-i\omega t} dt \tag{S73}$$

Through this approach we are now dealing with a signal that is continuous and aperiodic, spanning from $-\infty$ to $+\infty$. The Fourier integral is commonly used in analytical formulations, and we often use Fourier methods to treat signals (or data) that are aperiodic and discrete (note that the fact that a function needs to converge in the given interval in order to calculate its Fourier transform [7] may create difficulties in the analysis of viscoelasticity and in the calculation of frequency-dependent material parameters [9]). To treat such signals (data) a different type of transform from the Fourier family is used, namely the *Discrete Fourier Transform,* defined as:

$$\mathfrak{F}\{f[k]\} = \frac{1}{N} \sum_n f[n] e^{\frac{2\pi i n k}{N}} \tag{S74}$$

for which various calculation algorithms exist, such as the *Fast Fourier Transform (FFT*).

Before moving on, it is beneficial to discuss the frequency domain where Fourier transforms reside. The frequency domain is a complex domain, which is one-dimensional in the case of the Fourier transform. Every point in the frequency domain corresponds to a complex exponential function or, equivalently, a sine-cosine pair. The function in the frequency domain (upon application of the Fourier

transform) is the summation of all these points in the frequency domain, each multiplied with an appropriate coefficient, which may be complex. The points in the frequency domain constitute an orthogonal basis to project our function, and an illustration is offered in Figure S3.

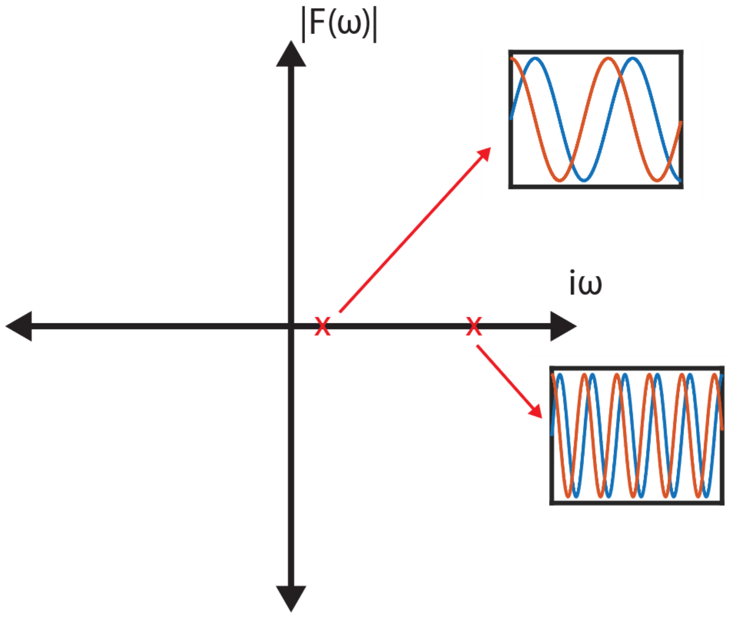

Figure S3: Illustration of different sine and cosine pairs corresponding to Fourier points in the frequency domain.

The inverse Fourier transform can be thought of as a summation of all the points in the frequency domain, each multiplied with its appropriate constant:

$$f(x) = \int_{-\infty}^{\infty} F(\omega)e^{-i\omega x}\, d\omega \tag{S75}$$

b. Modified Fourier Transform

As previously stated, for some functions the Fourier transform does not converge and/or there may be poles lying on the frequency axis, precluding the use of the transform and its inverse. An exponentially growing sinusoidal or a ramp function (such as those used in quasi-static AFM force-

distance experiments) are good examples of such cases. These limitations can be circumvented by altering the Fourier contour and/or by translating the frequency axis in the complex plane [7]. So far, we have only considered real frequencies with complex coefficients, which lie on the imaginary axis of the Fourier plane. However, we can add an imaginary constant offset ($i\beta_0$) to our frequency, which introduces a real component into our frequency domain, making it two-dimensional (see Figure S4):

$$\omega' = \omega + i\beta_0 \tag{S76}$$

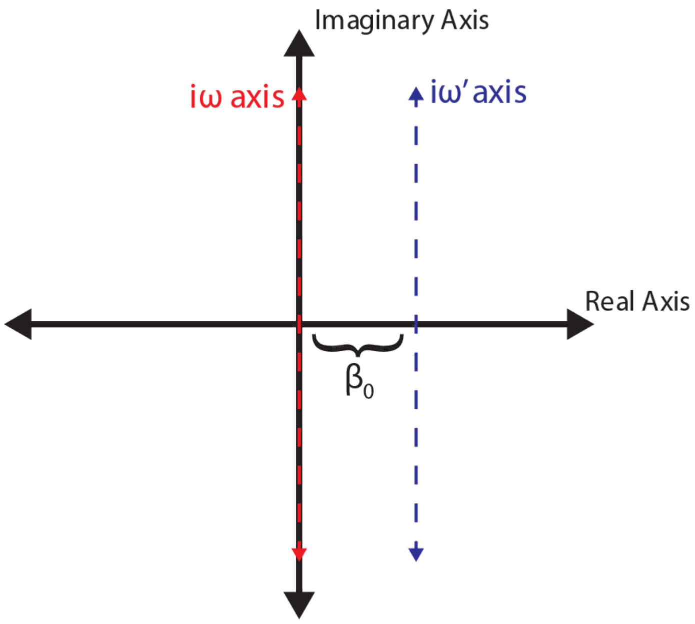

Figure S4: Comparison of the frequency domain of the Fourier transform and the modified Fourier transform. Introduction of a shifted frequency $\omega' = \omega + i\beta_0$ displaces the Fourier space (the imaginary axis) along the real direction. The new transformation based on ω' is called Modified Fourier Transform.

When we use our newly defined complex frequency (ω') in the Fourier transform, the new transformation is called Modified Fourier Transform:

$$F(\omega') = \int_{-\infty}^{\infty} f(t)e^{-i\omega' t}\,dt = \int_{-\infty}^{\infty} f(t)e^{-i(\omega+i\beta_0)t}\,dt = \int_{-\infty}^{\infty} f(t)e^{\beta_0 t}e^{-i\omega t}\,dt \tag{S77}$$

One may envision the Fourier transform as a representation of the original function through a continuum integration (summation) of sine-cosine pairs, and the modified Fourier transform as a representation of the function through an integration of exponentially decaying (or growing, depending on the chosen β_0 value) sine-cosine pairs.

To the readers who are accustomed to the Laplace transform, the modified Fourier transform should seem familiar. The main difference between the two transforms is that in the modified Fourier transform we have a *constant* real offset in the transformed space, while in the Laplace transform the entire real axis is available as a separate degree of freedom (in addition to the complex axis). One could, in fact, think of the Laplace transform as a generalized version of the modified Fourier transform, and many educational books on the subject start the discussion of the Laplace transform by first introducing the modified Fourier transform.

c. Laplace Transform

The Laplace transform is a very powerful tool used in engineering and applied science for treating differential equations. In fact, the theory of viscoelasticity and in particular the rheological approach to it, which is the main focus of this work, is derived using this transform in many sources. However, since it is an analytical tool defined for continuous functions, it is not possible to directly use it to analyze experimental data or measured signals, which are discrete in nature. Equation S78 defines the *s*-space variable used in Laplace methods and Equation S79 defines the Laplace transform:

$$s = \alpha + i\omega \tag{S78}$$

$$Ł\{f(t)\} = F(s) = \int_{0}^{\infty} f(t)e^{-ist}\,dt = \int_{0}^{\infty} f(t)e^{-i(\alpha+i\omega)t}\,dt \tag{S79}$$

Clearly, since the variable s consists of two variables, the Laplace space spans the entirety of the complex domain, and the transform of a function consists of a surface. Points in the s-domain correspond to sinusoidal functions, growing or decaying exponentials (in time), or sinusoidal functions with exponentially increasing or decreasing amplitude (in time). Reference [8] provides a useful illustration.

Comparison of Equations S79 and S73 shows that $\alpha = 0$ in the s-domain corresponds to the Fourier transform domain. Likewise, comparison of equations S77 and S79 shows that every (line) subdomain in the s-plane having a different value of α corresponds to the domain of a different modified Fourier transform of the function [8]. This is illustrated in Figure S5.

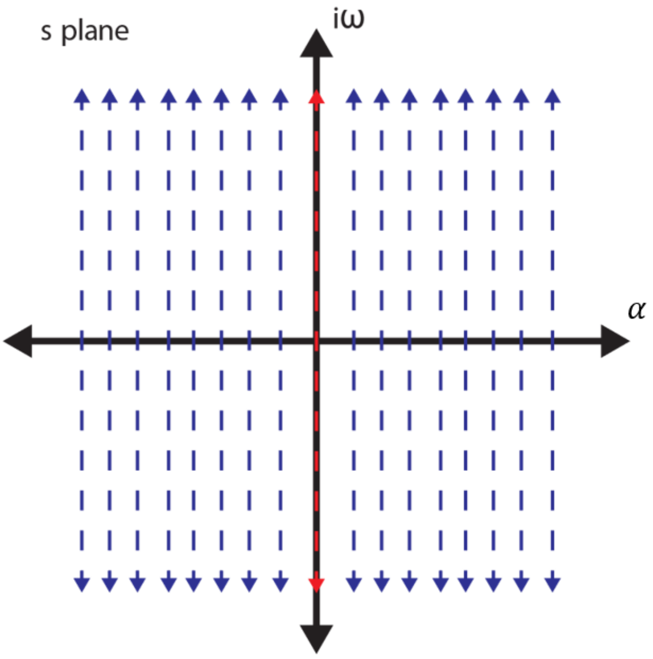

Figure S5: Illustration of the s-plane, which is equivalent to the complex plane. The imaginary axis, highlighted with a red dashed line is the domain of the Fourier transform. The other dashed lines, consisting of imaginary axes shifted by a real distance, correspond to the domains of different modified Fourier transforms of the function. The s-domain of the Laplace transform can be thought of as an infinite collection of modified Fourier transform domains, along with the Fourier transform domain, stitched together.

d. Z-Transform

The Z-transform enables us to handle discrete signals in a similar way as we would handle continuous functions using the Laplace transform [5]. Similar to our generalization in the previous section, we can think of the Z-transform domain as a collection of discrete Fourier transform domains. The Z-transform of a signal $f[n]$ is defined as:

$$\mathcal{Z}\{f[n]\} = F(z) = \sum_{n} f[n] z^{-n} \tag{S80}$$

where *z* is our complex variable, defined as:

$$z = re^{i\omega} \tag{S81}$$

whereas for the Laplace transform the variable *s* was defined as a sum of a real and an imaginary part as in Cartesian coordinates, the *z*-plane is defined in polar coordinates. However, regardless of this difference, we can still think of *z*- and *s*-planes as analogous planes. A comparison of both planes is provided in Figure 3 of the main manuscript and in the corresponding discussion in the text.

4- Fourier Transform of the Input-Output and the Misrepresentation of the System

As explained in the main manuscript, in an experiment with an unbounded input, such as the ramp function in quasi-static AFM, it is not possible to observe the steady state behavior of the material that would be the object of the Fourier transform. This is illustrated in Figure S6, where we have plotted the theoretical steady-state harmonic retardance of our model material, along with the retardance obtained from unit circle of the *z*-plane and the retardance obtained through the FFT. Clearly the latter two deviate significantly from the theoretical retardance.

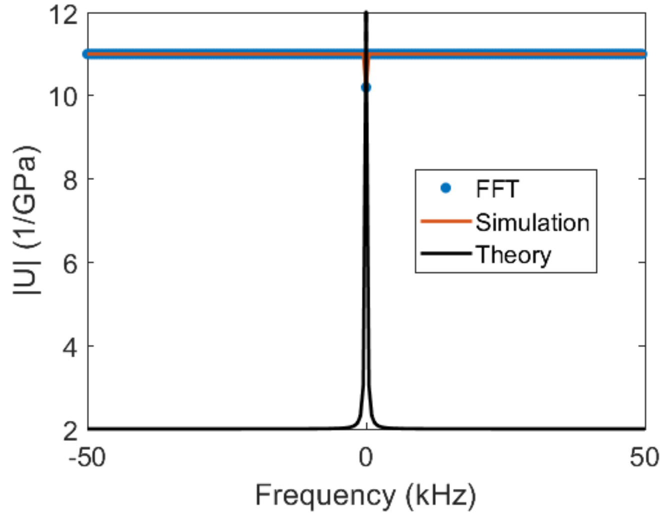

Figure S6: Comparison of the theoretical steady-state harmonic retardance of our model material (calculated using Equations 9 and S37) with the retardance obtained from the unit circle of the *z*-plane for our simulated experiment and the retardance calculated using the FFT. The FFT result was obtained by first calculating the FFT of the stress and the strain and then finding the transfer function between them by division. As discussed in the main manuscript, the information contained in the Fourier transform domain, accessed either from the unit circle of the *z*-plane or from the FFT algorithm for our model experiment, is not able to recreate the steady-state behavior of the system. Therefore, it is not possible for us to directly access the steady-state response of the material with a quasi-static force-distance curve experiment, which has an unbounded (ramp) input.

5- Data from Contact Mechanics Simulation

Figures S7 and S8 provide retardance profiles along the real axis of the *z*-plane and for different circles of the *z*-plane, similar to the data presented in Figures 6 and 7 of the main manuscript, respectively.

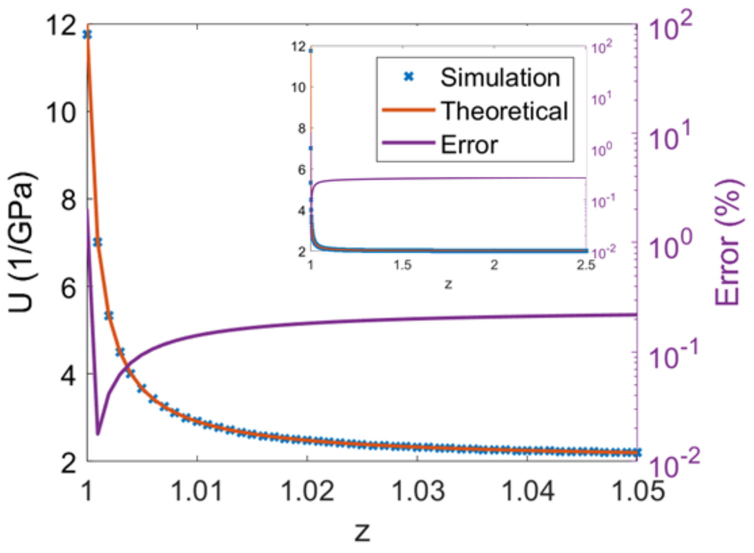

Figure S7: Retardance of the material obtained from the simulated experiment plotted along the real axis of the *z*-plane and compared to the theoretical retardance. The error is below 0.4% for the entire plot. As explained in the discussion of Figure 6 in the main paper, the real axis of the *z*-plane contains information about the material behavior at different time scales.

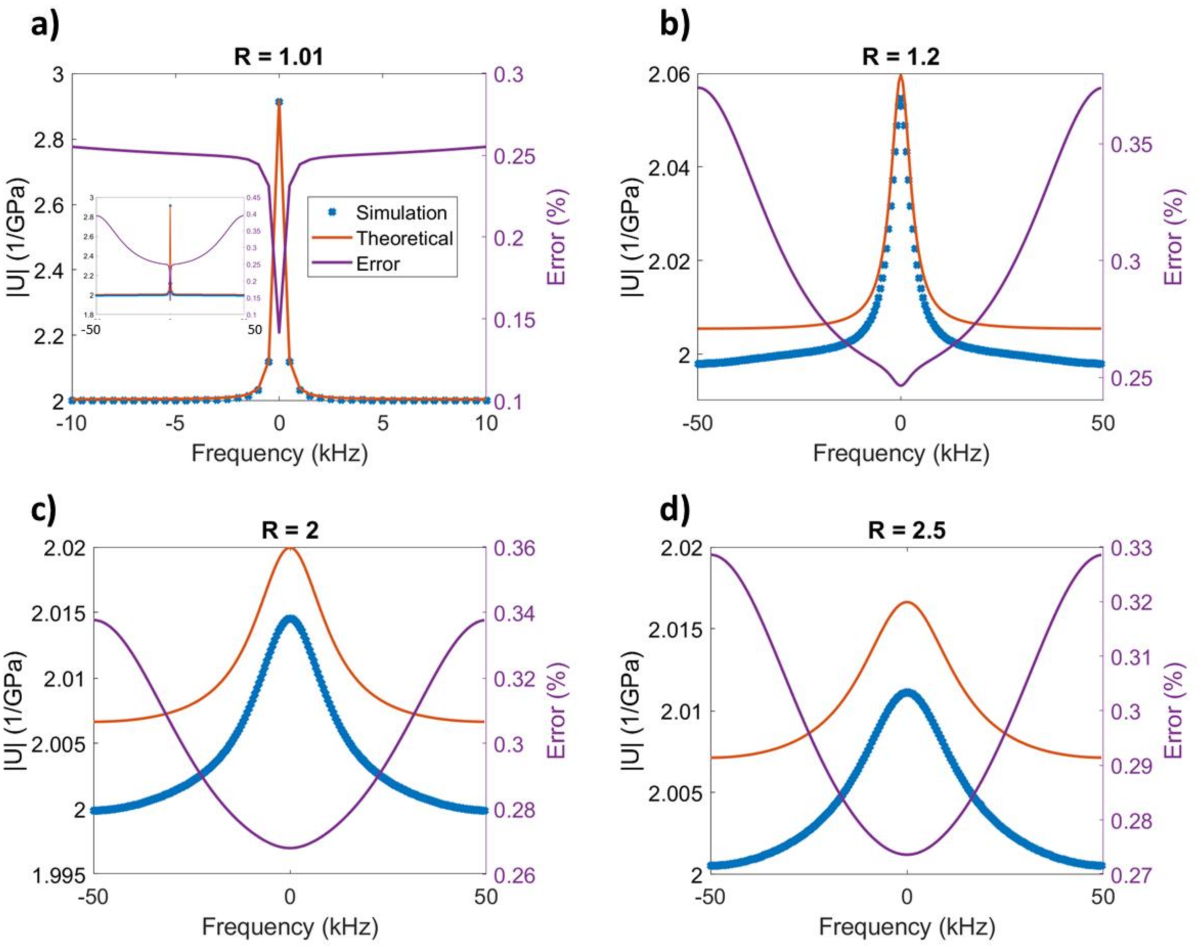

Figure S8: Retardance of the material plotted along circles of different radii of the *z*-plane. The radii of the circles are, respectively, 1.01, 1.2, 2, 2.5. As the plots illustrate, the retardance calculated from the simulated experiment closely matches the theoretical result with an error below 0.4%.

6- Analysis of Loss and Storage Viscoelastic Harmonic Functions and their Estimation from Modified Fourier Transforms

In this section we examine the relationship between the loss and storage behavior of the material in Fourier and Modified Fourier domains in order to illustrate the accuracy with which loss and storage functions can be estimated. The analysis is performed for both Generalized models considered in this work, namely the Generalized Voigt and the Maxwell-Wiechert models.

a) Generalized Voigt Model

As per Equation 10, the storage and loss compliances derive, respectively, from the real and imaginary parts of the retardance. Using the Generalized Voigt model, the retardance of a material without steady-state flow behavior can be written as:

$$U = J_g + \sum_n \frac{J_n}{\tau_n s + 1} \tag{S82}$$

We can express the steady-state behavior in the Fourier domain evaluating Equation S82 for $s = i\omega$, where $\alpha = 0$ (s is purely imaginary):

$$U(s = i\omega) = J_g + \sum_n \frac{J_n}{1 + i\tau_n \omega} \tag{S83}$$

Separating real and imaginary components we can immediately write the storage and loss compliances:

$$J' = J_g + \sum_n \frac{J_n}{1 + \tau_n^2 \omega^2} \tag{S84}$$

$$J'' = \sum_n \frac{J_n \omega \tau_n}{1 + \tau_n^2 \omega^2} \tag{S85}$$

Close inspection of Equations S84 and S85 shows that the storage compliance (equation S84) is a summation of Lorentzian functions, one per characteristic time, plus a constant glassy compliance. Lorentzian functions are well-known from harmonic motion dynamics, Cauchy distributions and many other different applications. The loss compliance has a similar shape, but instead of simple Lorentzian functions, we have a Lorentzian function multiplied by a frequency, which is also the independent variable. We can also re-arrange these compliance expressions into a more common Lorentzian expression:

$$J' = J_g + \sum_n J_n \frac{\frac{1}{\tau^2}}{\frac{1}{\tau^2} + \omega^2} \tag{S86}$$

$$J'' = \sum_n (J_n \tau_n)\omega \frac{\frac{1}{\tau^2}}{\frac{1}{\tau^2} + \omega^2} \tag{S87}$$

We see that each different Voigt unit contributes a Lorentzian function to the storage compliance. Furthermore, these functions are centered around the zero frequency and have a peak magnitude equal to their respective compliance. The width of the Lorentzian contribution decreases with increasing characteristic time. In fact, the half-width at half-maximum is equal to $1/\tau$ radians/second. In other words, the longer the characteristic time, the sharper the function becomes. Likewise, the shorter the characteristic time, the bandwidth of the Lorentzian increases and encompasses more frequencies.

A similar observation can be made for the loss compliance. The width of the Lorentzian contribution also decreases with increasing characteristic time. However, per equation S87, the Lorentzian function is multiplied with frequency, so the dependence of the peak width on the characteristic time is not symmetric. The decay of the Lorentzian is faster than the increase (ramping) of the frequency that multiplies it, and the expression converges to zero at very high frequencies:

$$\lim_{\omega \to \infty} J'' = \lim_{\omega \to \infty} \sum_n \frac{J_n \omega \tau_n}{1 + \tau_n^2 \omega^2} = \frac{\infty}{\infty} \tag{S88}$$

$$\lim_{\omega \to \infty} J'' = \lim_{\omega \to \infty} \sum_n \frac{\frac{d(J_n \omega \tau_n)}{d\omega}}{\frac{d(1 + \tau_n^2 \omega^2)}{d\omega}} = \lim_{\omega \to \infty} \sum_n \frac{J_n \tau_n}{2\tau_n^2 \omega} = 0 \tag{S89}$$

Since the high-frequency limit of the loss compliance is zero and the function is lopsided towards the left, greater energy loss takes place in the lower frequency ranges. Because of the frequency ramp, the function does not have its maximum value at zero, but rather at its characteristic frequency $1/\tau_n$ radians/second:

$$J_n'' = \frac{J_n \omega \tau_n}{1 + \tau_n^2 \omega^2} \tag{S90}$$

$$\frac{dJ_n''}{d\omega} = -\frac{J_n\tau_n(\tau_n^2\omega^2 - 1)}{(1 + \tau_n^2\omega^2)^2} \tag{S91}$$

$$\left(\frac{dJ_n''}{d\omega} = 0\right) @ \left(\omega = \omega_{max} = \frac{1}{\tau_n}\right) \tag{S92}$$

Furthermore, the height of the amplitude peak of the loss compliance is proportional to both the compliance of the corresponding Voigt unit and its characteristic time. Thus, greater loss occurs for larger characteristic times and vice-versa.

We now investigate how the above result would change when using a modified Fourier transform. We can write down a Modified Fourier transform of the retardance by simply evaluating its Laplace transform along a vertical line on the s-plane, that is evaluating Equation S82 at $s = \alpha_0 + i\omega$, where α_0 is a constant and the frequency is our variable:

$$U(s = \alpha_0 + i\omega) = J_g + \sum_n \frac{J_n}{1 + \tau_n(\alpha_0 + i\omega)} = J_g + \sum_n \frac{J_n}{(1 + \tau_n\alpha_0) + i\tau_n\omega} \tag{S93}$$

We can now separate the real and imaginary components by rationalization of the denominator:

$$U(s = \alpha_0 + i\omega) = J_g + \sum_n \frac{J_n(1 + \tau_n\alpha_0 - i\tau_n\omega)}{(1 + \tau_n\alpha_0)^2 + \tau_n^2\omega^2} \tag{S94}$$

$$J'_{\alpha_0} = J_g + \sum_n \frac{J_n + J_n\tau_n\alpha_0}{(1 + \tau_n\alpha_0)^2 + \tau_n^2\omega^2} \tag{S95}$$

$$J''_{\alpha_0} = \sum_n \frac{J_n\tau_n\omega}{(1 + \tau_n\alpha_0)^2 + \tau_n^2\omega^2} \tag{S96}$$

For an easier comparison, we can write Equations S95 and S96 in Lorentz-Cauchy distribution form, just as Equations S86 and S87:

$$J'_{\alpha_0} = J_g + \sum_n \frac{J_n}{1 + \tau_n\alpha_0} \frac{\frac{(1 + \tau_n\alpha_0)^2}{\tau_n^2}}{\frac{(1 + \tau_n\alpha_0)^2}{\tau_n^2} + \omega^2} \tag{S97}$$

$$J''_{\alpha_0} = \sum_n \omega \frac{J_n\tau_n}{(1 + \tau_n\alpha_0)^2} \frac{\frac{(1 + \tau_n\alpha_0)^2}{\tau^2}}{\frac{(1 + \tau_n\alpha_0)^2}{\tau_n^2} + \omega^2} \tag{S98}$$

We see that the above expressions follow the same trends as the storage and loss compliance given in equations S86 and S87. Furthermore, the glassy compliance portion of the storage compliance remains the same for the Fourier and the modified Fourier transform. Due to these similarities, it is possible to make an approximation of the storage and loss compliances using the real and imaginary parts of the modified Fourier transform result. However, there are also some differences that need to be considered. First, the width of the Lorentzian contributions of the individual Voigt units differs, with the Lorentzian contributions of the modified Fourier transforms having wider peaks by a factor of $(1 + \tau_n \alpha_0)$. This factor also changes the amplitude of the individual contributions, which are reduced by a factor of $(1 + \tau_n \alpha_0)$. This factor may be negligible in cases where the characteristic time of the unit is very small or, more feasibly, when the modified Fourier time constant α_0 is small (i.e., when the chosen vertical line on the *s*-plane is sufficiently close to the imaginary axis, which represents the Fourier transform). In the *z*-domain, the factor $(1 + \tau_n \alpha_0)$ becomes $\left(1 + \frac{\tau_n \ln(r)}{\Delta t}\right)$ due to the unit conversion discussed in the derivation of rheological models for the *z*-domain. One final, but very important takeaway is that the position of the local maxima of the loss compliance in the modified Fourier domain are given by:

$$\left(\frac{dJ''_{\alpha_0}}{d\omega} = 0\right) @ \left(\omega = \omega_{max} = \frac{(1 + \tau_n \alpha_0)}{\tau_n}\right) \quad \text{(S99)}$$

This corresponds to $\frac{1 + \frac{\tau_n \ln(r)}{\Delta t}}{2\pi \tau_n}$ in the z-domain in units of Hz. Therefore, one can also pinpoint the characteristic times of the material using the imaginary component of a modified Fourier transform.

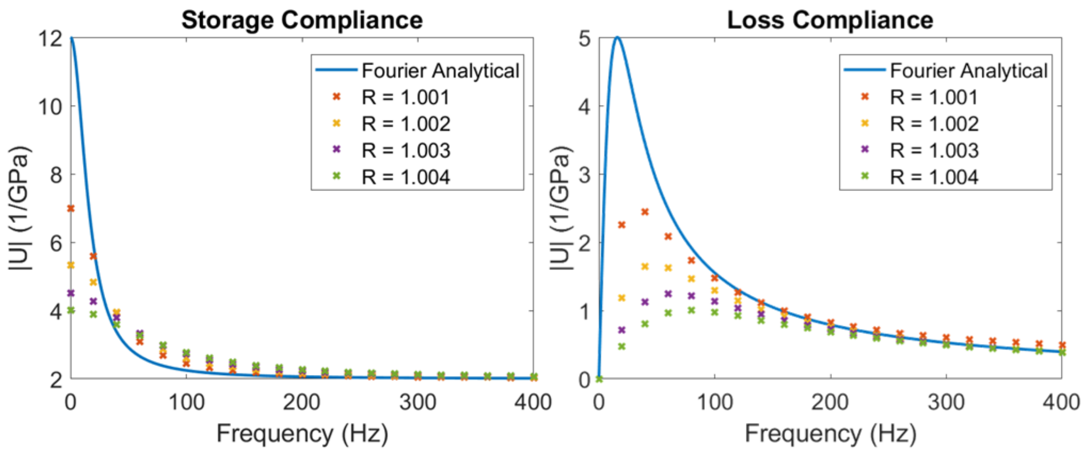

Figure S9: Comparison between the analytical loss and storage compliances for our model material and their estimation from a modified Fourier transform evaluated from the Z-transform.

b) Maxwell-Wiechert Model

The loss and storage moduli for the Maxwell-Wiechert model can be obtained from the real and imaginary components of the retardance, which can be written for a material that does not experience steady-state flow behavior as:

$$Q = G_g - \sum_n \frac{G_n}{\tau_n s + 1} \quad \text{(S100)}$$

We now evaluate this expression at $s = i\omega$ (where $\alpha = 0$) to focus on the material's steady-state behavior:

$$Q(s = i\omega) = G_g - \sum_n \frac{G_n}{1 + i\tau_n \omega} \quad \text{(S101)}$$

Next, we separate the real and imaginary components and write the storage and loss moduli, respectively:

$$G' = G_g - \sum_n \frac{G_n}{1 + \tau_n^2 \omega^2} \quad \text{(S102)}$$

$$G'' = \sum_n \frac{G_n \omega \tau_n}{1 + \tau_n^2 \omega^2} \tag{S103}$$

We note that the storage modulus (Equation S102), takes the shape of a summation of one Lorentzian function per characteristic time being subtracted from a constant glassy modulus G_g. Since $G_g = G_e + \sum G_n$, where G_e is the rubbery modulus, at zero frequency the storage modulus is equal to the rubbery modulus. As the frequency grows, the Lorentzian functions approach zero and the storage modulus approaches the glassy modulus. For the loss modulus we have a similar expression, but with the Lorentzian function being multiplied by the frequency. We can, again, re-arrange these expressions in a way that their Lorentzian functions become more obvious:

$$Q' = G_g - \sum_n G_n \frac{\frac{1}{\tau^2}}{\frac{1}{\tau^2} + \omega^2} \tag{S104}$$

$$Q'' = \sum_n (G_n \tau_n) \omega \frac{\frac{1}{\tau^2}}{\frac{1}{\tau^2} + \omega^2} \tag{S105}$$

Each different model unit contributes a Lorentzian to the storage modulus and the function peaks are centered around the zero frequency, with the peak magnitude being equal to their respective moduli. The peak width is inversely proportional to the characteristic time and the half-width at half-maximum equals $1/\tau$ radians/seconds.

For the loss modulus the width of the Lorentzian contribution is inversely proportional to the corresponding characteristic time, but since the function is multiplied with frequency, the loss function is again asymmetric (greater energy loss happens in the lower frequency ranges):

$$\lim_{\omega \to \infty} Q'' = \lim_{\omega \to \infty} \sum_n \frac{G_n \omega \tau_n}{1 + \tau_n^2 \omega^2} = \frac{\infty}{\infty} \tag{S106}$$

$$\lim_{\omega \to \infty} Q'' = \lim_{\omega \to \infty} \sum_n \frac{\frac{d(G_n \omega \tau_n)}{d\omega}}{\frac{d(1 + \tau_n^2 \omega^2)}{d\omega}} = \lim_{\omega \to \infty} \sum_n \frac{G_n \tau_n}{2\tau_n^2 \omega} = 0 \tag{S107}$$

The loss modulus reaches its maximum value at the characteristic frequency $1/\tau_n$ radians/seconds:

$$Q_n'' = \frac{G_n \omega \tau_n}{1 + \tau_n^2 \omega^2} \tag{S108}$$

$$\frac{dQ_n''}{d\omega} = -\frac{G_n \tau_n (\tau_n^2 \omega^2 - 1)}{(1 + \tau_n^2 \omega^2)^2} \tag{S109}$$

$$\left(\frac{dQ_n''}{d\omega} = 0 \right) @ \left(\omega = \omega_{max} = \frac{1}{\tau_n} \right) \tag{S110}$$

Furthermore, the peak amplitude of the loss modulus contribution is proportional both to the modulus of the unit and to its characteristic time.

As for the Voigt model, we can write down a modified Fourier transform of the retardance by evaluating its Laplace transform along a vertical line in the s-plane. Setting $s = \alpha_0 + i\omega$ in Equation S100, where α_0 is a constant and the frequency is our variable, we obtain:

$$Q(s = \alpha_0 + i\omega) = G_g - \sum_n \frac{G_n}{1 + \tau_n(\alpha_0 + i\omega)} = G_g - \sum_n \frac{G_n}{(1 + \tau_n \alpha_0) + i\tau_n \omega} \tag{S111}$$

We now separate real and imaginary parts:

$$Q(s = \alpha_0 + i\omega) = G_g - \sum_n \frac{G_n(1 + \tau_n \alpha_0 - i\tau_n \omega)}{(1 + \tau_n \alpha_0)^2 + \tau_n^2 \omega^2} \tag{S112}$$

$$Q'_{\alpha_0} = G_g - \sum_n \frac{G_n + G_n \tau_n \alpha_0}{(1 + \tau_n \alpha_0)^2 + \tau_n^2 \omega^2} \tag{S113}$$

$$Q''_{\alpha_0} = \sum_n \frac{G_n \tau_n \omega}{(1 + \tau_n \alpha_0)^2 + \tau_n^2 \omega^2} \tag{S114}$$

Finally, we recast the expressions in the form of Lorentz-Cauchy distributions:

$$Q'_{\alpha_0} = G_g - \sum_n \frac{G_n}{1 + \tau_n \alpha_0} \frac{\frac{(1 + \tau_n \alpha_0)^2}{\tau^2}}{\frac{(1 + \tau_n \alpha_0)^2}{\tau^2} + \omega^2} \tag{S115}$$

$$Q''_{\alpha_0} = \sum_n \omega \frac{G_n \tau_n}{(1 + \tau_n \alpha_0)^2} \frac{\frac{(1 + \tau_n \alpha_0)^2}{\tau^2}}{\frac{(1 + \tau_n \alpha_0)^2}{\tau^2} + \omega^2} \tag{S116}$$

Not surprisingly, the real and imaginary components of the modified Fourier transform follow the same trends as the previously developed storage and loss modulus expressions, with the glassy modulus remaining unchanged. Here again it is possible to approximate the storage and loss moduli using the real and imaginary parts of the modified Fourier transforms, with the same considerations as for the previous model. Specifically, the peak widths of the Lorentzian contributions are different, with the modified Fourier transforms functions being wider by a factor of $(1 + \tau_n \alpha_0)$. Furthermore, the amplitude of the individual peaks decreases by $(1 + \tau_n \alpha_0)$. This factor may or may not be significant, depending on the value of the characteristic time of the unit and the value of α_0. The factor $(1 + \tau_n \alpha_0)$ again changes into $\left(1 + \frac{\tau_n \ln(r)}{\Delta t}\right)$ for the z-transform. As we would expect in light of the previous model's derivations, the positions of the local amplitude maxima of Equation S98 and S116 are the same and equal to $\frac{(1+\tau_n \alpha_0)}{\tau_n}$, which also correspond to $\frac{1+\frac{\tau_n \ln(r)}{\Delta t}}{2\pi\tau_n}$ Hz.

REFERENCES

(1) Roylance, D. Engineering Viscoelasticity. Massachusetts Institute of Technology: MIT OpenCouseWare 2001, pp 1–38

(2) Tschoegl, N. W. *The Phenomenological Theory of Linear Viscoelastic Behavior*; Springer Berlin Heidelberg: Berlin, Heidelberg, 1989. doi:10.1007/978-3-642-73602-5

(3) López-Guerra, E. A. Analytical Developments for the Measurement of Viscoelastic Properties with the Atomic Force Microscope, The George Washington University, 2018

(4) López-Guerra, E. A.; Eslami, B.; Solares, S. D.; López-Guerra, E. A.; Eslami, B.; Solares, S. D. *J. Polym. Sci. Part B Polym. Phys.* **2017**, *55* (10), 804–813. doi:10.1002/polb.24327

(5) Oppenheim, A. V.; Schafer, R. W. *Digital Signal Processing*; Pretince-Hall: New Jersey, 1975

(6) McClellan, J. H.; Schafer, R. W.; Yoder, M. A. *Signal Processing First*; Pearson\Prentice Hall: Upper Saddle River, NJ, 2003

(7) Kusse, B. R.; Westwig, E. A. *Mathematical Physics*; Wiley, 2006. doi:10.1002/9783527618132

(8) Smith, S. W. *The Scientist & Engineer's Guide to Digital Signal Processing*, 1st ed.; California Technical Publishing: San Diego, 1997

(9) Evans, R. M. L.; Tassieri, M.; Auhl, D.; Waigh, T. A. *Phys. Rev. E* **2009**, *80* (1), 012501. doi:10.1103/PhysRevE.80.012501
